# PTSD subtypes and their underlying neural biomarkers: a systematic review

**DOI:** 10.1017/S0033291725001229

**Published:** 2025-05-22

**Authors:** Chen Zhang, Shilat Haim-Nachum, Neal Prasad, Benjamin Suarez-Jimenez, Sigal Zilcha-Mano, Amit Lazarov, Yuval Neria, Xi Zhu

**Affiliations:** 1 Department of Psychiatry, Columbia University Medical Center, New York, NY, USA; 2 New York State Psychiatric Institute, New York, NY, USA; 3 School of Social Work, Tel Aviv University, Tel Aviv, Israel; 4 Department of Neuroscience, University of Rochester, Rochester, NY, USA; 5 Department of Psychology, University of Haifa, Haifa, Israel; 6 School of Psychological Sciences, Tel-Aviv University, Tel Aviv, Israel; 7 Department of Bioengineering, University of Texas at Arlington

**Keywords:** biotypes, data-driven approaches, neuroimaging, posttraumatic stress disorder, subtypes

## Abstract

Posttraumatic stress disorder (PTSD) is a heterogenous disorder with frequent diagnostic comorbidity. Research has deciphered this heterogeneity by identifying PTSD subtypes and their neural biomarkers. This review summarizes current approaches, symptom-based group-level and data-driven approaches, for generating PTSD subtypes, providing an overview of current PTSD subtypes and their neural correlates. Additionally, we systematically assessed studies to evaluate the influence of comorbidity on PTSD subtypes and the predictive utility of biotypes for treatment outcomes. Following the PRISMA guidelines, a systematic search was conducted to identify studies employing brain imaging techniques, including functional magnetic resonance imaging (fMRI), structural MRI, diffusion-weighted imaging (DWI), and electroencephalogram (EEG), to identify biomarkers of PTSD subtypes. Study quality was assessed using the Strengthening the Reporting of Observational Studies in Epidemiology (STROBE) guidelines. We included 53 studies, with 44 studies using a symptom-based group-level approach, and nine studies using a data-driven approach. Findings suggest biomarkers across the default-mode network (DMN) and the salience network (SN) throughout multiple subtypes. However, only six studies considered comorbidity, and four studies tested the utility of biotypes in predicting treatment outcomes. These findings highlight the complexity of PTSD’s heterogeneity. Although symptom-based and data-driven methods have advanced our understanding of PTSD subtypes, challenges remain in addressing the impact of comorbidities and the limited validation of biotypes. Future studies with larger sample sizes, brain-based data-driven approaches, careful account for comorbidity, and rigorous validation strategies are needed to advance biologically grounded biotypes across mental disorders.

## Introduction

Posttraumatic stress disorder (PTSD) is a complex psychiatric disorder that may develop following exposure to traumatic events, as defined by the Diagnostic and Statistical Manual of Mental Disorders (DSM), such as threatened death, severe injury, or sexual violence. PTSD manifests with significant heterogeneity, with research indicating over 636,120 possible symptom combinations (Galatzer-Levy & Bryant, 2013). Additionally, PTSD is often comorbid with other disorders, such as depression, anxiety, or substance use. This comorbidity influences its clinical manifestation, with patients with multiple comorbidities experiencing more severe symptoms (Blanchard, Buckley, Hickling, & Taylor, 1998), higher risk of suicidal behavior (Oquendo et al., 2003), greater impairments in neurocognitive functioning (Nijdam, Gersons, & Olff, 2013), and poorer treatment outcomes (Green et al., 2006).

In recent years, much progress has been made in exploring brain structures and functions to better understand the heterogeneity of PTSD. Most studies have focused on comparing subjects with PTSD to normal control (NC) participants to identify disorder-specific biomarkers (Ben-Zion et al., 2020; Goodkind et al., 2015). While these studies laid the groundwork for identifying major neural biomarkers that differ between PTSD from NC, the high heterogeneity within PTSD and its frequent diagnostic comorbidity have led to inconsistent neuroimaging findings. Consequently, the field has faced challenges in reliably translating knowledge from neuroimaging into clinical practice (Casey et al., 2013).

**Figure 1.**
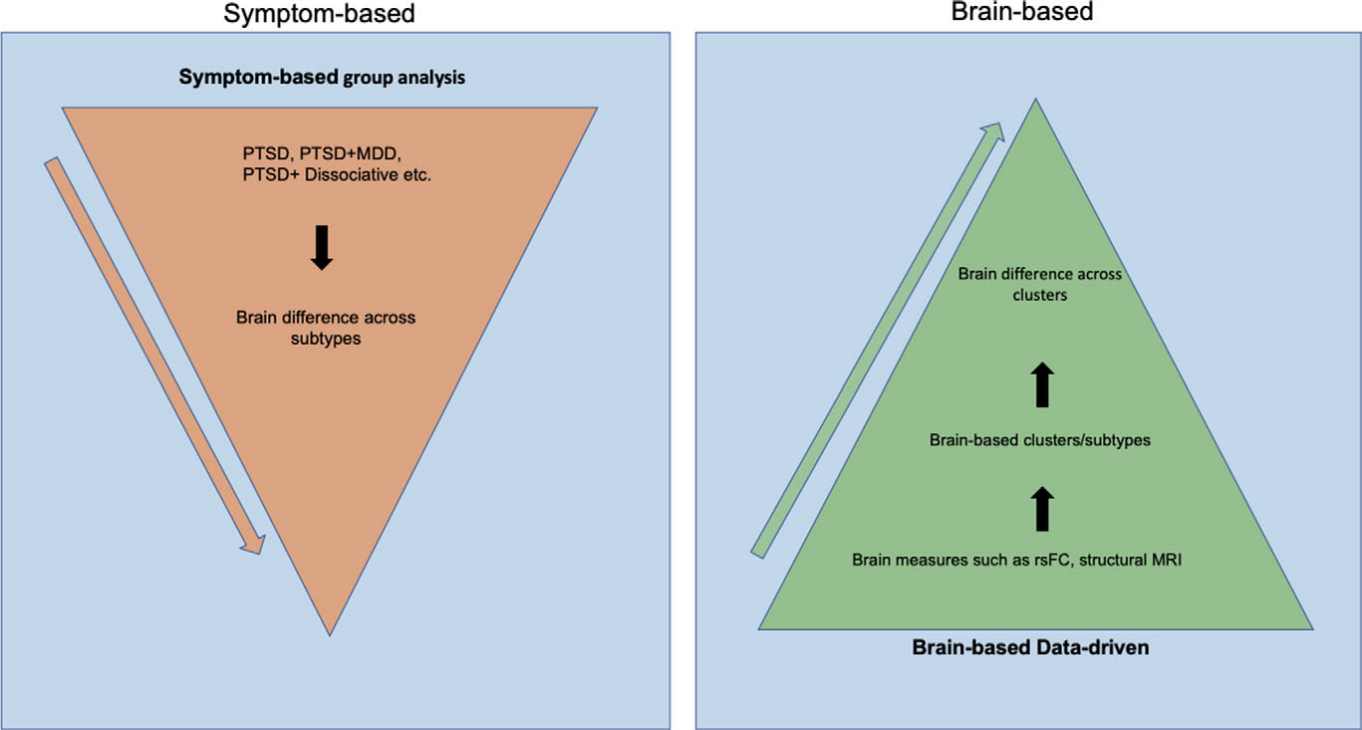
This figure illustrates the two approaches to understanding the subtypes of PTSD, including the DSM-based top-down method, symptom-based group analysis, and brain-based data-driven method. The arrow indicates the order of each step in generating subtypes. The orange triangles illustrate the steps to generate PTSD subtypes using a symptom-based group analysis approach. The green triangle on the right describes the steps in creating subtypes using a brain-based data-driven approach.

To address this complexity, recent efforts have increasingly focused on identifying more homogeneous PTSD subtypes and their underlying brain mechanisms (biotypes). A *subtype* refers to a more homogeneous subgroup within a disorder that shares specific symptom patterns/characteristics, potentially reflecting distinct etiologies, neural mechanisms, or treatment responses (Jiang et al., 2024). Two main approaches have been commonly used to identify PTSD subtypes: symptom-based group-level approaches, and brain-based data-driven approaches (Figure 1).

*Symptom-based group-level approaches* typically employ a univariate group-level analysis, or supervised machine learning analysis, to compare two or more PTSD subtypes, defined by specific PTSD symptoms or DSM classifications. For example, prior research used this approach by comparing PTSD with and without dissociative symptoms (Harricharan et al., 2016); PTSD with and without impaired executive functioning (Begic & Jokic-Begic, 2007; Guffanti et al., 2016; Nicholson et al., 2019; Zoellner, Pruitt, Farach, & Jun, 2014); individuals with PTSD who responded to treatment to those who did not; and PTSD with and without comorbid depression (Zhu et al., 2017). These studies have provided insights into the neural underpinnings of homogenous clinical clusters. However, top-down approaches often oversimplify PTSD’s complex symptomatology, potentially missing the nuanced interplay of symptoms, especially in comorbid cases. The DSM-5, for instance, encompasses a total of 628 distinct symptoms, with 36.8% of symptoms recurring across multiple diagnoses (Stein et al., 2014). This symptom overlap is even more pronounced among highly comorbid disorders like PTSD and major depressive disorder (MDD).

To address the heterogeneity in diagnoses, *Brain-based data-driven approaches* have been introduced. These approaches aim to identify PTSD subtypes based on biomarkers (biotypes) utilizing neuroimaging data only, without the use of DSM classification or symptoms. This is based on the assumption that individuals with similar symptomatology or DSM diagnoses may still differ in underlying biological alterations (Insel et al., 2010). This assumption may also explain the low effect sizes found in treatment trials, as treatments are conducted among patients with the same diagnosis with potential neurobiological heterogeneity (Drysdale et al., 2017). Thus, identifying biotypes in individuals with PTSD using brain-based data-driven approaches could provide insight into patient-specific neurobiological mechanisms and better treatment match (Cuijpers et al., 2016). Neuroimaging studies have attempted to characterize data-driven biotypes underlying specific psychiatric disorders (Fair, Bathula, Nikolas, & Nigg, 2012; Van Dam et al., 2017) and explored these biotypes’ utility in predicting treatment outcomes. For example, (Drysdale et al., 2017; Etkin et al., 2019) identified biotypes defined by distinct dysfunctional connectivity using a large multisite MDD sample; these biotypes predicted which patients would profit from transcranial magnetic stimulation (TMS). Together, these studies show that data-driven approaches are promising and capable of discovering brain-based biotypes that differ in clinically meaningful ways.

Despite numerous studies investigating PTSD subtypes through the above two approaches, comorbidity and validation remain challenges. Comorbidity often complicates the interpretation of its underlying neurobiology. For example, PTSD+MDD shows hypo-connectivity between the DMN and the SN, while PTSD alone shows hyperconnectivity in the same regions (Kaiser, Andrews-Hanna, Wager, & Pizzagalli, 2015; Nicholson et al., 2020; Xu et al., 2019; Yuan et al., 2019). This variability suggests that the observed heterogeneity in PTSD could be attributed to inadequate clarity regarding comorbid conditions, characterized by both a lack of formal exclusions and comprehensive assessments in research investigations.

Furthermore, validation strategies – such as reproducibility and utility assessments – are essential to ensure the validity and reproducibility of the generated subtypes (Brucar, Feczko, Fair, & Zilverstand, 2023). Reproducibility focuses on confirming whether the identified biotypes can be consistently replicated across different datasets, methodologies, or populations. This can be assessed through internal validation, which tests the reproducibility within the same dataset, and external validation, which evaluates the consistency of findings across different datasets. Assessing the utility of biotypes is equally important, as it refers to the application of biotypes in relation to secondary measures such as clinical outcomes, behavioral measures, and treatment responses. In clinical settings, rigorous testing of biotype utility is crucial for advancing the field of personalized medicine in PTSD care, ultimately leading to more efficient and effective treatments that are tailored to the individual patient. The inconsistent application of validation strategies in a data-driven approach may contribute to the heterogeneous neurobiological findings.

Thus, the first aim of this systematic review is to summarize current methods for identifying PTSD subtypes, including symptom-based group-level approaches and brain-based data-driven approaches, and to explore how these methods influence subtype definitions. This review addresses existing literature gaps, as no prior reviews have systematically integrated both approaches. The second aim is to systematically compare and evaluate studies to understand how differences in comorbidity inclusion and validation strategies might influence findings and conclusions regarding PTSD subtypes. Our review focuses on determining whether these studies have rigorously tested the utility of biotypes, including assessing how the biotypes relate to clinical and behavioral measures and their ability to improve patient treatment responses.

## Methods

### Study identification and key words

This systematic review was completed according to the Preferred Reporting Items for Systematic Reviews and Meta-Analyses (PRISMA) guidelines (Parums, 2021). A comprehensive search was completed using PubMed, Library of Congress, LISTA, and Web of Science Core Collection, from inception up to February 2023. Reference lists from relevant studies and reviews were examined to identify eligible studies. For details on selection of trauma-related keywords, please refer to Supplementary Material. Studies were included if they met one or more of the following criteria: (1) Participants of the study must be over the age of 18 and complete the neuroimaging data collection process; (2) participants must have PTSD or partial PTSD; (3) assessment of PTSD was conducted with a standardized measure according to a diagnostic classification system such as DSM-5 (First, 2013), International Classification of Diseases (ICD-11) (World Health Organization, 1992); (4) more than one PTSD subtype is identified in the PTSD population; (5) in English; and (6) published in peer-reviewed journals. Studies were excluded if they were non-experimental papers such as reviews or meta-analyses, non-MRI studies, case reports, animal studies, or non-English papers.

### Study screening and data extraction

Two reviewers (CZ and NP) screened and reviewed the study abstracts independently using Covidence (Covidence systematic review software, Veritas Health Innovation, Melbourne, Australia. Available at www.covidence.org). For more detail on the specific processes, please refer to Supplementary Material.

### Biotype reproducibility strategies

Biotype utility strategies include internal validation and external validation. Internal validation refers to validation strategies that evaluate the stability of the cluster solution within one dataset across different splits, or via internal cluster information such as validity indices. External validation refers to optimizing model generalizability by testing the model on an independent, external dataset.

### Biotype utility strategies

Biotype utility strategies refer to the clinical utility of biotypes, they can be assessed via cross-sectional and longitudinal data. For cross-sectional data, studies should evaluate the association between the identified biotypes and clinical (e.g., mild and severe symptoms) and behavioral profiles (e.g., cognitive control, reward processing) that were not used in the initial biotype discovery. These studies could also build a classification model to evaluate the ability to classify among biotypes or between each biotype and HC group, with the aim of improving DSM-based diagnostics. For longitudinal data, studies should evaluate whether the identified biotypes exhibit differences in treatment outcome and efficacy.

## Results

**Figure 2.**
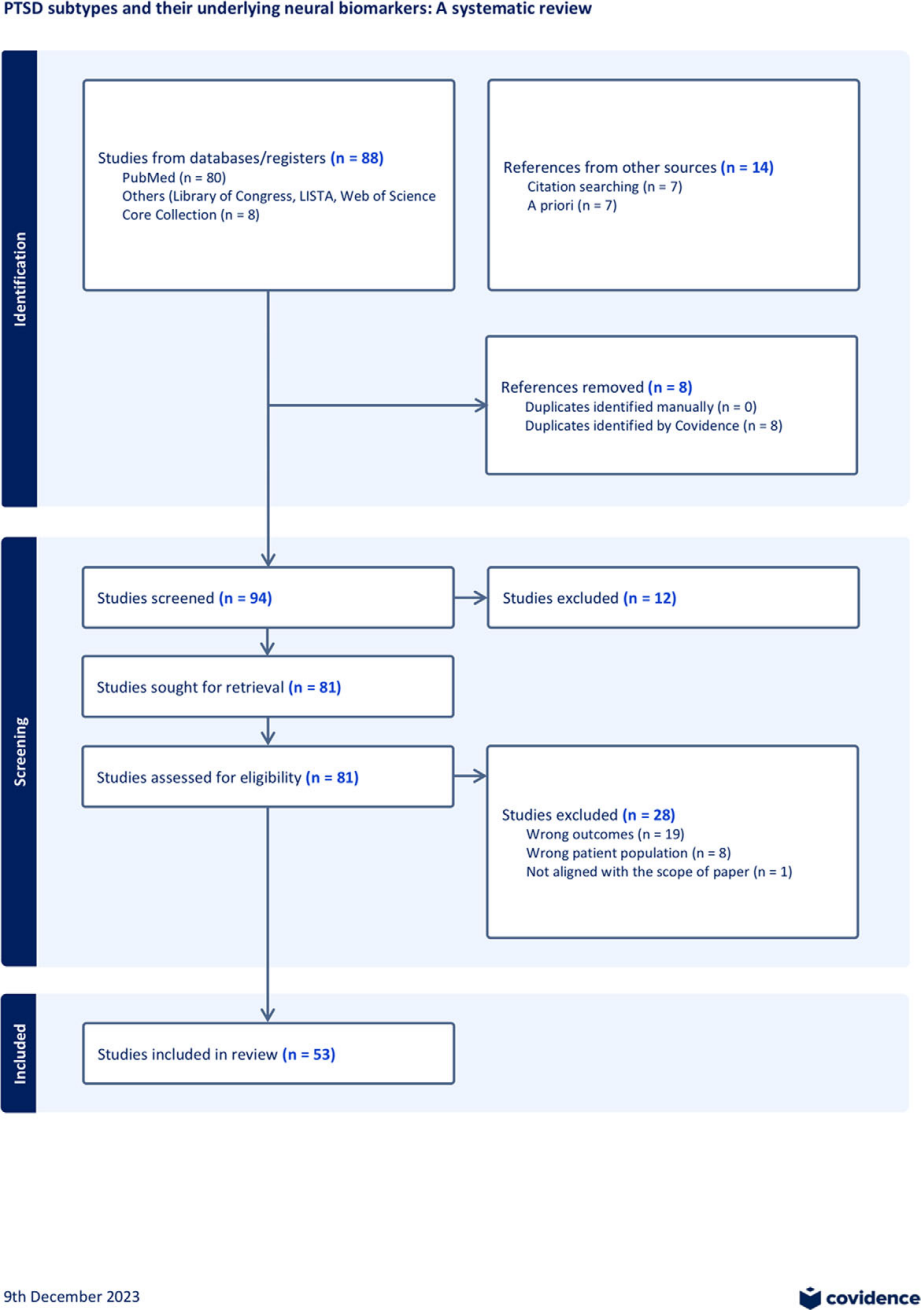
Flowchart for study inclusion. Initial literature search was conducted via PubMed, Library of Congress, LISTA, Web of Science Core Collection using keywords (refer to Supplementary Material Methods). Additional literature was added based on reviewing reference lists and a priori knowledge. The PRISMA review methods were used to evaluate the research articles. A final number of 53 studies were included. For additional details, refer to Supplementary Material.

The results section synthesizes findings from studies on PTSD subtypes, addressing the heterogeneity challenge by evaluating network alterations, different approaches to generating subtypes, and their implications for treatment outcomes. The review includes 53 studies and highlights variations in sample sizes, trauma types, and neuroimaging modalities. Additionally, the section emphasizes the differences between symptom-based and data-driven methods in identifying subtypes, providing insight into their utility for personalized treatment strategies in PTSD. A flowchart is provided in Figure 2, and a summary of the included studies is provided in Supplementary Table 1. Detailed study summaries and further data are available in Supplementary Materials.

Due to differences in data analysis methods and imaging modalities, and a comparatively small proportion of the bottom-up data-driven analysis methods versus the top-down analysis methods, a systematic review that summarizes the current field of knowledge in PTSD subtypes was conducted rather than a meta-analysis.

**Figure 3.**
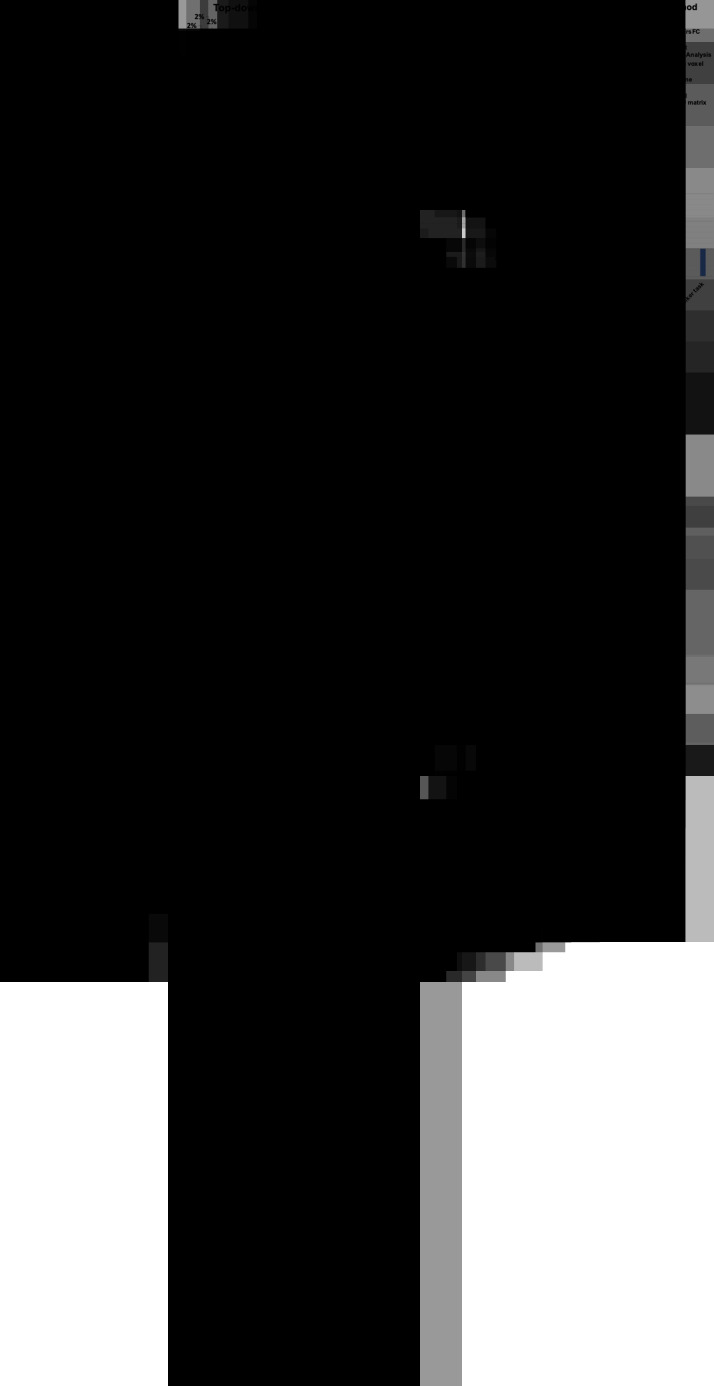
This figure illustrates the study overview through the following four figures. (a) This graph presents the percentage of available studies using each of the two identified approaches: symptom-based group analysis and brain-based data-driven approach. (b) This graph summarizes the percentage of selected studies that investigated different categories of PTSD subtypes using a top-down approaches; 10 subtypes of PTSD have been under investigation, with the dissociative subtype being the most researched subtype as 40% of studies investigated dissociative subtypes. (c) This graph illustrates the percentage of studies that utilized each type of neuroimaging modality. (d) This graph provides an overview of the percentage of studies that used different imaging feature selection methods. (e) This graph provides an overview of the percentage of studies that investigated each type of trauma. (f) This graph is an overview of the number of literature with the respective sample populations; most studies include a sample size of 66 to 156. (g) This chart provides an overview of the types of tasks used by the task-based fMRI studies. The current review study found a total of 13 fMRI task-based studies; the figure demonstrates that the most used task is the emotional picture task, followed by Go/NoGo task and the Script-driven imagery task. The chart reveals that the tasks used are diverse, each task is used by one to three studies.

Of the 55 studies, 46 (84%) used a symptom-based group-level approach (Figure 3a). Within this group, seven studies (13%) used symptom-based supervised machine learning to distinguish different PTSD subtypes, and 39 (71%) used group-level univariate analysis to identify distinct biomarkers among biotypes. Among the 46 studies, two studies used both group-level univariate analysis and supervised machine learning analysis. The remaining nine (16%) studies employed a brain-based data-driven approach. Across all 55 studies, sample sizes varied significantly, with most of the studies sampling between 66 and 156 patients (26 studies, 47%, mean: 115), followed by studies sampling between 156 and 381 patients (17 studies, 31%, mean: 215), and studies sampling between 21 and 66 patients (12 studies, 22%, mean: 47) (Figure 3f). An overview of investigated trauma types revealed most studies (18 studies, 33%) did not specify trauma type. This was followed by veteran combat trauma, which included 17 studies (31%), and interpersonal violence, childhood trauma, and post-911 veterans, which included 11 studies (20%) (Figure 3e). Regarding neuroimaging data modalities, 30 studies (55%) used measures derived from resting-state fMRI (rs-fMRI), ten studies (18%) used task-based fMRI, four studies (7%) used structural magnetic resonance imaging (s-MRI), three studies (5%) used diffusion tensor imaging (DTI), one study (2%) used EEG, and eight studies (15%) used two or more modalities (Figure 3c).

### Symptom-based group-level approaches

Forty-six studies examined the biotypes by comparing PTSD with a subtype of PTSD based on DSM criteria or symptoms-based classification (Figure 3b). The most frequently studied subtype in this category was the dissociative subtype (DS) (19 studies, 41%), followed by executive function (EF) (five studies 11%). Six studies (13%) focused on symptom dimensions subtype (hyperarousal, avoidance, reexperiencing, fear, and dysphoria). Five studies (11%) compared PTSD with comorbidity and PTSD. Four studies (9%) focused on comparing responders and non-responders at baseline following psychotherapy response. Two studies (4%) compared PTSD with suicide ideation (SI) with PTSD. Two studies (4%) compared PTSD with complex-PTSD (c-PTSD). One study compared PTSD with early life stress (ELS) subtype (2%). One study (2%) compared PTSD with disinhibition subtype, and one study (2%) compared PTSD with self-blame subtype. For the review, summaries of DS and data-driven subtypes are reported in the results section. Given the limited number of studies in the following subtypes: EF, symptom-based, comorbidity, psychotherapy response, SI, cPTSD, ELS, disinhibition, and self-blame, a detailed review of each study is in Supplementary Material.

The **dissociative subtype** **(DS)** is the most studied subtype of PTSD. Expanding on the review by Lotfinia, Soorgi, Mertens, & Daniels (2020), which used structural and task-based fMRI data, the current literature focuses on a network-based approach to examine dissociative symptoms. Notably, 88% of the studies investigating the DS subtype have focused on resting-state functional connectivity (rsFC). Current findings reveal greater ventromedial PFC (vmPFC) connectivity within anterior default mode network (aDMN), increased within-salience network (SN) connectivity in PTSD+DS (Nicholson et al., 2016, 2019, 2020). Moreover, consistent alteration was reported in the following between-network connectivity in PTSD+DS: increased pDMN-SN (Harricharan et al., 2020; Nicholson et al., 2015; 2020; Sierk et al., 2021), increased DMN-brainstem (Harricharan et al., 2016; Nicholson et al., 2019; Olive et al., 2018; Thome et al., 2019), and decreased executive control network (ECN)-brainstem (Harricharan et al., 2017; Olive et al., 2018).

Other top-down based subtypes (e.g., executive function subtype, SI, ELS, symptom-based, comorbidity, psychotherapy response) reveal alterations in DMN, ECN, SN, limbic network (LN), ventral attention network (VAN), and visual network. DMN, ECN, and SN exhibit deficits across all subtypes.

### Data-driven subtypes approach

The brain-based data-driven method employs clustering algorithms to generate subtypes using solely brain-based data, resulting in two to four subtypes in these studies. Nine studies in our review utilized brain-based data-driven approaches, relying on measures such as s-MRI, rs-fMRI, task-based fMRI, and EEG to parse PTSD heterogeneity.

Various unsupervised clustering methods were employed in these nine studies, including latent class analysis (Zilcha-Mano et al., 2022), k-means clustering (Ahrenholtz et al., 2021), hierarchical clustering (Zhao et al., 2018), cluster identification via connectivity kernels (CLICK) clustering algorithm (Maron-Katz et al., 2020; Sharan, Maron-Katz, & Shamir, 2003), and Group Iterative Multiple Model Estimation (GIMME) (Gates & Molenaar, 2012; Stout et al., 2021; Strigo, Spadoni, & Simmons, 2022). Seven studies included either trauma-exposed healthy controls (TEHC) or both TEHC and PTSD in the clustering analysis, while the other two studies only included PTSD or PTSD and comorbidity. Five studies selected priori regions of interest (ROIs) to reduce feature dimensions for clustering analysis. Two to four data-driven clusters were identified in these nine studies. Six out of nine studies carried out internal validation; four out of these six studies only used cross-validation within the entire dataset. Two out of the six studies carried out external validation, in which clustering analysis was done at each site (Stevens et al., 2021; Zhao et al., 2018), or was done separately on the discovery cohort and replication cohort (Stevens et al., 2021). One study evaluated the utility of the identified biotypes in prolonged exposure therapy (Stout et al., 2021). All studies utilized a sample size of <200 participants.

Currently, biomarkers identified through a data-driven approach lack common consensus. Individual studies vary in the number of clusters identified, as well as the differentiating biomarker. Two clusters were identified using structural MRI, the thalamus and rostral middle frontal gyrus differentiate the two clusters (Zilcha-Mano et al., 2022), while two clusters created using rs-fMRI differ through visual and sensorimotor connectivity in another investigation (Maron-Katz et al., 2020). In contrast, task-based studies generated three to four clusters within PTSD (Ahrenholtz et al., 2021; Stevens et al., 2021). Despite the data-driven approach’s promising prospect, the current results of data-driven studies are highly heterogeneous, thereby challenging to draw conclusions. A detailed biomarker summary can be found in Supplementary Material.

### Comorbidity

Out of 53 studies, 15 studies excluded other Axis-I psychiatric diagnosis, 13 studies excluded participants with concurrent/history of developmental disorder, 30 studies excluded any substance dependence and/or abuse, 32 studies excluded participants with history of traumatic brain injury, loss of consciousness, or neurological disorders, 42 studies excluded history or concurrent psychotic/bipolar disorders, and 22 studies included, but not limited to, the use of anxiety/depressive symptom subscales such as HAMD, BDI, HRSD, BAI, and CGI. Out of the 15 studies that excluded axis-I, five of them investigated comorbidity by comparing PTSD only with comorbid PTSD+MDD. Out of 39 studies that did not exclude axis-I psychiatric comorbidity, only two of these studies specifically tested the sensitivity of the main results by including comorbidity as covariates (Jagger-Rickels et al., 2021, 2022).

Upon further examination, we identified patterns of comorbidities’ influence on biomarkers. For example, one study that did not exclude participants with brain injury history reported that PTSD+DS exhibited increased rsFC between right ECN and right lateral OFC comparing to PTSD-DS (Nicholson et al., 2020), potentially due to the absence of overlapping impact of brain injury history, whereas another study that excluded participants with the history of brain injury, identified increased connectivity within salience network, cerebellar network, and hubs of cortico-basal ganglia-thalamic loops within PTSD+DS, but not in PTSD-DS (Nicholson et al., 2019). Studies that excluded Axis-I comorbidities tended to focus on homogeneous PTSD profiles, leading to clearly defined neurobiological markers. For instance, studies excluding comorbid MDD reported more distinct neural alterations within DMN, ECN, and SN (Bryant et al., 2021; Li et al., 2020), potentially due to the absence of overlapping depressive symptoms. Conversely, studies that included comorbid MDD often reported an interaction of these networks in relation to the striatal-subcortical network (Sierk et al., 2021; Yuan et al., 2019; Zhu et al., 2017), possibly reflecting the influence of depressive symptoms on PTSD neurobiological profile.

### Validation strategies

For group-level analysis, no validation strategy was applied. Among the nine machine learning studies, five examined cluster reproducibility through either only internal validation or both internal and external validation. All five studies conducted internal validations through repeated iterations, including leave-one-subject-out-cross-validation (LOSOCV) or 10-fold cross-validation within the dataset (Ahrenholtz et al., 2021; Li et al., 2022; Maron-Katz et al., 2020; Stevens et al., 2021; Zhang et al., 2021; Zilcha-Mano et al., 2022). Two of the nine studies used internal and external validation strategies; in both studies, external validation involved replicating the implemented clustering strategy in an independent but related dataset (Stevens et al., 2021; Zhang et al., 2021). Five of the nine studies investigated cluster utility; three of these identified potential treatment targets through studying the distinct traits/profiles of each subtype (Ahrenholtz et al., 2021; Li et al., 2022; Zilcha-Mano et al., 2022). Among the three studies, two assessed the utility of identified biotypes in prolonged exposure therapy using longitudinal follow-up data (Stout et al., 2021; Zhang et al., 2021), one using both clinical and behavioral assessment and another using only clinical assessments to investigate subtype utility (Maron-Katz et al., 2020; Stevens et al., 2021). Lastly, only one study examined subtype’s follow-up clinical response to different types of treatment (Zhang et al., 2021).

## Discussion

Our review summarized PTSD subtypes and their associated brain biomarkers. The symptom-based group-level approach remains dominant, comprising 81% of the studies. Among these studies, seven studies have leveraged machine learning techniques to elucidate the multivariate relationships between brain regions, two studies applied external validation strategies, and five studies investigated cluster utility. Lastly, only two studies tested the sensitivity of the main results by including comorbidity as a covariates. This underlines a critical gap in utilizing machine learning approaches to leverage larger datasets, integrate multimodal imaging data, employ rigorous cross-validation and validation strategies, and provide individual-level prediction, which is important for translating research into clinical practice.

The symptom-based top-down approach reveals nine categories of PTSD subtypes, while studies utilizing a data-driven bottom-up approach typically find two to four PTSD subtypes based on brain data. Among subtypes, the dissociative subtype of PTSD emerged as an area of focus, comprising 41% of the studies. Biomarkers within SN, DMN, VAN, ECN, cerebellar, visual network, and brainstem network were identified by different imaging modalities (e.g., grey matter volume [GMV], rs-FC, mALFF). This review highlights a lack of consistent directionality pattern identification within these networks, which may be due to small sample sizes, variations in ROIs, and differences in data processing. However, eight subtypes consistently show functional and structural alterations in the DMN, SN, and/or ECN. Specifically, Two subtypes (DS, symptom-based) reported DMN and SN alterations (Grupe, Wielgosz, Davidson, & Nitschke, 2016; Harricharan et al., 2020; Nicholson et al., 2015; 2020; Stumps et al., 2021), ELS subtype reported only DMN hub disruption (Salminen et al., 2019), two subtypes (cPTSD, disinhibition) reported only ECN disruptions (Herzog et al., 2019; Sadeh et al., 2015), and three other subtypes (symptom-based, psychotherapy-response, suicidality) reported ECN and SN alterations (Bomyea, Stout, & Simmons, 2019; Stumps et al., 2021; van Rooij, Kennis, Vink, & Geuze, 2016; Zhutovsky et al., 2019).

As the only subtype recognized and listed by the DSM-5, the DS subtype appears to be predominantly favored in the current investigation using symptom-based approaches to understand PTSD heterogeneity (Ross et al., 2018). Symptom-based approaches understand heterogeneity through DSM-5, which adopts a symptom-based, diagnostic category, rather than a dimensional approach, as proposed by the Research Domain Criteria (RDoC). This reinforces the dominance of the DS subtype in PTSD research and limits the exploration of other PTSD subtypes, such as the emotional or cognitive subtypes (e.g., self-blame, disinhibition, or executive functioning difficulties), and restricts the broader understanding of PTSD heterogeneity. Future research should explore a broader range of PTSD symptomatology beyond dissociation following the RDoC approach. Specifically, additional research could investigate whether other symptom clusters – such as anhedonia, hyperarousal, and negative mood – may also correspond to distinct neurobiological profiles. Expanding the scope of biotype identification to incorporate these additional symptom dimensions will help ensure a more comprehensive understanding of symptom-level PTSD subtypes.

Notably, a major limitation in the reviewed studies is the lack of comorbidity consideration in their analyses. Only five top-down studies compared PTSD with comorbid MDD with PTSD, while only one study investigated PTSD+AUD. Another two studies tested the sensitivity of the main results by including comorbidity as covariates (Jagger-Rickels et al., 2021; 2022). In addition, 40 out of 55 studies did not actively exclude possible existing or lifetime axis-I diagnoses. Thus, comorbidity may have complicated efforts to classify each disorder as a single distinct phenotype (Neria, 2021), identify its clinical course, and understand its biomarkers (Stein et al., 2014). For example, research suggests that PTSD+MDD exhibits striatal-subcortical network alterations affecting fear and reward processing (Yuan et al., 2019; Zhu et al., 2017). This finding differs from studies that excluded Axis I disorders, highlighting the impact of co-occurring psychiatric conditions on neurobiological heterogeneity. While excluding lifetime or current Axis I diagnoses can help isolate PTSD-specific neural signatures, strict exclusion criteria may limit generalizability in PTSD. Future studies can consider a more balanced approach involving covariate adjustments and sensitivity analyses for comorbid conditions in statistical models. Moreover, to address heterogeneity, the RDoC initiative offers a transdiagnostic strategy for understanding shared neural mechanisms across highly comorbid disorders, such as PTSD, depression, and anxiety disorders (Insel, 2014). For example, research has shown that transdiagnostic treatments targeting common neurobiological mechanisms were associated with improvements in comorbid mood and anxiety disorders (McEvoy, Norton, & Peter, 2009). To address this issue, future studies could incorporate symptom subscales, such as anxiety/depressive mood subscales, to capture dimensional symptom overlap between PTSD and comorbid disorders. Identifying data-driven, biologically informed biotypes in PTSD and its comorbid disorders and mapping them onto dimensional symptoms using RDoC approach, rather than traditional diagnostic categories could enhance our understanding of pathophysiology and facilitate precision medicine approaches. While this review does not focus specifically on RDoC, it highlights the potential of symptom-based, data-driven approaches to offer a more nuanced understanding of PTSD (Ben-Zion et al., 2020). Moving forward, symptom-based, data-driven approaches present new methodological avenues for understanding the neurobiological basis of highly comorbid disorders, overcoming the limitations of a symptom-based group-level approach.

Another limitation in the reviewed studies is that 87% of the studies used a single imaging modality, and 69% of the studies’ sample size is small (*n* < 156), limiting the ability to identify robust PTSD biotypes. Multimodal biotypes with diverse neural disturbance profiles and clinical characteristics remain largely unexplored across highly comorbid disorders in large samples. Future studies can incorporate multimodal neuroimaging to improve biotype validation and transferability by repeating clustering across diverse neuroimaging modalities (Maron-Katz et al., 2020; Zhang et al., 2021). Studies also used different imaging feature selection methods, which may contribute to variability in findings. Among the 55 studies, 11 studies used ROI-based analysis, 18 used seed-based analysis, and 12 used whole-brain voxel-wise analysis (Figure 3d). Whole-brain voxel-wise analysis methods provide an unbiased approach but are more susceptible to noise. In contrast, some studies used ROI-based methods focusing on hypothesis-driven regions, which may enhance sensitivity for detecting specific effects but may overlook broader neural patterns relevant to PTSD subtypes. Future studies should leverage large-scale multimodal neuroimaging datasets, such as ENIGMA consortium, to ensure generalizability and reliability across diverse populations, while ensuring reproducibility across different imaging feature selection methods.

We also reviewed the validation strategies used in the data-driven studies. Validation strategies are necessary to enhance the transferability and applicability of subtyping methods in precision medicine. Our review found that five out of nine data-driven studies implemented cluster reproducibility, and only two investigated cluster utility of the PTSD biotypes. Consistent with recent literature on data-driven studies (Brucar, Feczko, Fair, & Zilverstand, 2023), only 5% of the 38 studies validated the utility of identified biotypes in clinical treatment samples. The methodological inconsistency of validation strategies and variations within subject exclusion criteria create diverse PTSD subtypes, posing the challenge of drawing direct comparisons and conclusions, thus influencing subtypes’ cluster utility and clinical translation. Future studies should develop prospective validation pipelines to enhance the transferability and applicability of biotypes; standardize computational and statistical validation pipelines to improve replicability and comparability across studies; utilize transfer learning to enhance the predictive power and stability of biotypes across different populations, datasets, and imaging modalities; and establish open-access benchmark datasets to facilitate cross-study replication and comparison of biotypes across studies.

**Figure 4.**
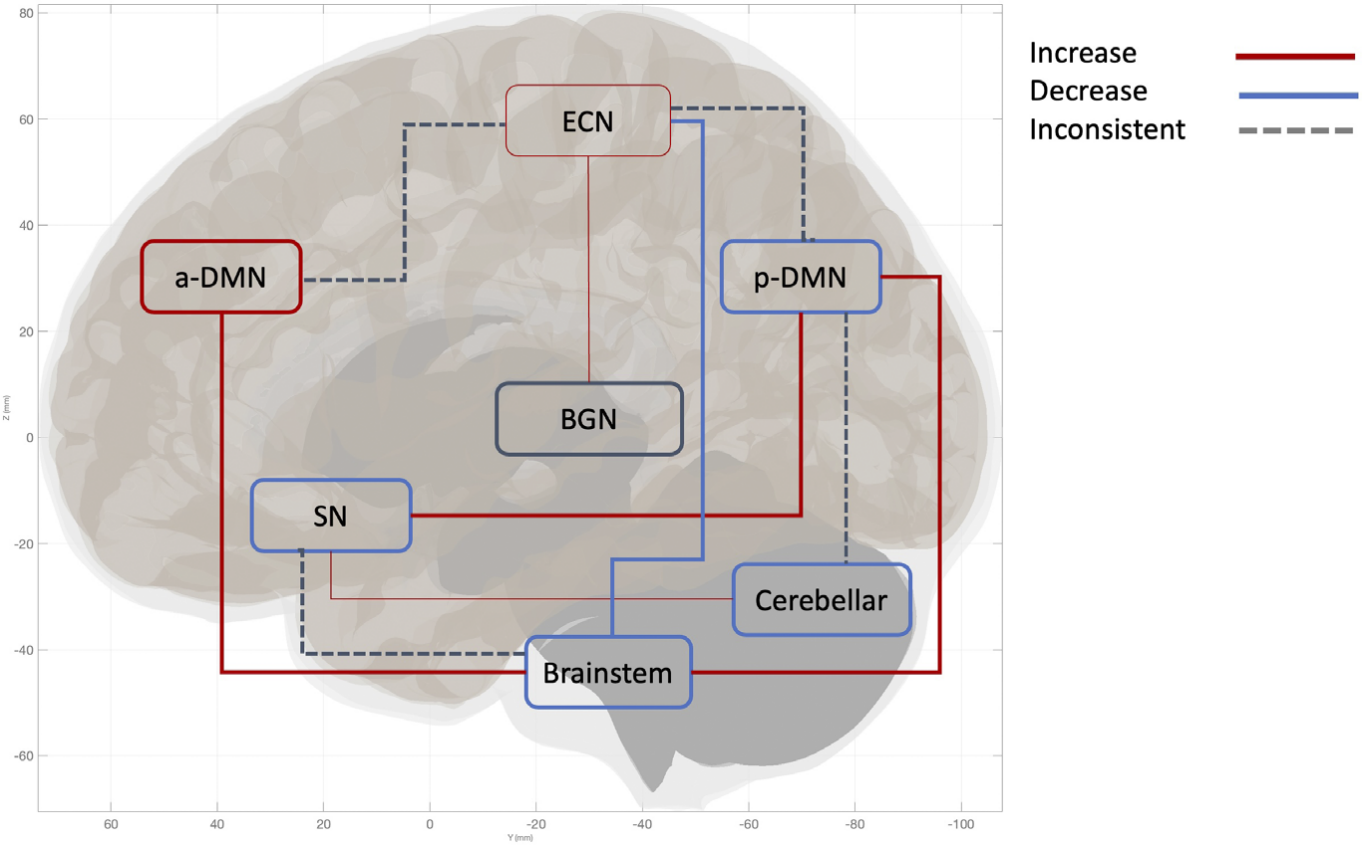
This figure illustrates the neural network alterations within the dissociative subtypes, specifically the difference in the directionality of connectivity within PTSD+DS vs. PTSD-DS. Thick lines indicate two or more studies found consistent directionality of connectivity. The narrow line indicates the directionality of the connectivity is implicated by one study. Arrows illustrate the pattern of between-network connectivity; the square around the network names represents the pattern of within-network connectivity. Increased between-network connectivity is represented by a red line and observed between the following networks: pDMN-brainstem, SN-pDMN, aDMN-Brainstem, ECN-BGN. Increased within-network connectivity observed in aDMN, ECN. Decreased network connectivity is represented by the blue line, observed between the following networks: ECN-Brainstem. Decreased within-network connectivity was found in the following networks: pDMN, SN, Cerebellar network, and Brainstem. An inconsistent network pattern, represented by a grey dotted line, was found between the following networks: SN-Brainstem, Cerebellar-pDMN, ECN-aDMN, ECN-pDMN. Inconsistent within-network connectivity found in BGN. The network is defined as the following brain hubs. pDMN: Posterior DMN, including PCC, precuneus, TPJ. aDMN: anterior DMN, including vmPFC. SN: salience network, including amygdala, insula. ECN: executive control network, including DLPFC, frontal pole, anterior cingulate cortex.

In the contrary, symptom-based subtype utility has been explored in top-down studies through the psychotherapy subtype, involving longitudinal prediction (i.e., the identification of baseline biotypes that can predict future psychotherapy outcomes over time). Specifically, six out of 45 top-down studies defined subtypes by treatment response, making these relevant for understanding PTSD heterogeneity and assessing treatment utility. Four out of the six psychotherapy subtypes investigated biomarkers underlie differential treatment responses towards trauma-focused cognitive-behavioral therapy (TF-CBT) (Bryant et al., 2021; Korgaonkar et al., 2021; van Rooij, Kennis, Vink, & Geuze, 2016; Zhutovsky et al., 2019), one out of these four TF-CBT studies investigated both TF-CBT and EMDR (van Rooij et al., 2016). Two out of the six studies investigated prolonged exposure (PE) (Etkin et al., 2019; Zilcha-Mano et al., 2020), and one of these two PE studies examined both PE and transcranial magnetic stimulation (TMS) (Etkin et al., 2019). All six studies are longitudinal in design and identified baseline biomarker that predicts post-treatment response (Bryant et al., 2021; Korgaonkar et al., 2021; van Rooij et al., 2016; Zhutovsky et al., 2019). One study investigated the change-based predictive biomarkers. There is a lack of investigation of medication utility in relation to PTSD subtypes. To advance treatment options, future research should explore diverse modalities, such as medication, TMS, or different forms of psychotherapy. In addition, future studies could implement biotype-based stratification in treatment trials to assess whether distinct biotypes predict differential treatment response. For example, DMN-altered subtypes may benefit from belief-altering treatment (cognitive-processing therapy) due to its role in the abnormal acquirement of self-related belief in PTSD (Baldassano, Hasson, & Norman, 2018; Yeshurun, Nguyen, & Hasson, 2021). SN-altered Subtypes (e.g., symptom-based and suicidality subtypes) may benefit from treatments targeting emotional disruption induced by threat detection, such as EMDR, PE, or emotion-focused therapy (Schimmelpfennig, Topczewski, Zajkowski, & Jankowiak-Siuda, 2023; Szeszko & Yehuda, 2019). ECN-altered subtypes (e.g., cPTSD and disinhibition) may exhibit responsivity to TMS (Edinoff et al., 2022; Martin-Signes, Cano-Melle, & Chica, 2021), given its role in top-down cognitive control of co-existing conflicting information to regulate emotion (Bush, Luu, & Posner, 2000; Carter, Botvinick, & Cohen, 1999). DS subtypes may benefit from bodily based treatment such as somatic psychotherapy due to its between-network alteration of the brainstem, which is involved in self-awareness, bodily perception, and proprioception integration (Benarroch, 2018; Blanke & Arzy, 2005; Edlow et al., 2023; Harricharan et al., 2016, 2017; Olive et al., 2018; Tsunematsu, Patel, Onken, & Sakata, 2020). Future research should investigate the clinical validity and efficacy of these biomarkers through longitudinal clinical trials (Figure 4).

## Conclusion

Limited by DSM, symptom-based group analysis prevails in understanding PTSD subtypes and overlooks non-DS subtypes, comorbidity’s impact, hindering subtype clarity. The current methodology lacks effective validation strategies, particularly in data-driven studies, limiting reliability. To advance PTSD diagnosis and treatment, research must embrace data-driven approaches for biotypes, consider comorbidity’s influence, and implement robust validation methods for reliable clinical application.

The lack of personalized medicines contribute to trial-and-error recovery process, as well as a systemic financial burden (Davis et al., 2022; Green et al., 2006; Kalra, Kamath, Trivedi, & Janca, 2008). Aiming to advance mental disorder diagnosis, further investigation requires larger sample sizes, diverse nosological categories, and imaging modalities. Future research should focus on the application of PTSD subtypes and treatment individualization, moving towards a transdiagnostic approach which could reduce financial burdens, reduce trial-and-error processes to enhance treatment efficiency and improve overall population wellness.

## Supporting information

Zhang et al. supplementary material Zhang et al. supplementary material

## Abbreviations

AAL: Anatomical Labeling
ACC: Anterior Cingultae Cortex
BAI: Beck Anxiety Inventory
BDI: Beck Depression Inventory
BGN: Basal-ganglia Network
BLA: Basolateral Amygdala
BNST: Bed Nucleus of the Stria Terminals
BT: Biotype
CADSS: State Identity Dissociation Symptoms
CAPS-IV: Clinical Administered PTSD Scale
CDS: Cambridge Depersonalization Scale
CEN: Central Executive Network
CGI: Clinical Global Impression scale
CMA: Centromedial Amygdala
CPT: Cognitive Processing Therapy
cPTSD: Complex PTSD
CTQ: Child Trauma Questionnaire
dACC: Dorsal ACC
DAN: Dorsal Attention Network
DID: Dissociative Identity Disorder
dlPFC: Dorsolateral PFC
DMN: Default-mode Network
dmPFC: Dorsal Medial PFC
DTI: Diffusion Tensor Imaging
ELS: Early Life Stress
EPIC: Evolving Partitions to Improve Classification
FA: Fractional Anisotropy
FPCN: Fronto-parietal Control Network
GAD: Generalized Anxiety Disorder
GIMME: Group Iterative Multiple Model Estimation
HC: Healthy Control
HRSD: Hamilron Rating Scale for Depression
ICA: Independent Component Analysis
LCA: Latent Class Analysis
LN: Limbic Network
LOSOCV: Leave-one-subject Out Cross-validation
mALFF: mean Amplitude Low-frequency Fluctuations
MDD: Major Depression Disorder
MDI: Multiscale Dissociation Inventory
MDI: Major Depression Inventory
ML: Machine Learning
MRI: Magnetic-resonance Imaging
NAcc: Nucleus Accumbens
OCD: Obsessive Compulsive Disorder
OFC: Orbitofrontal Cortex
PAG: Periaqueductal Gray
PCC: Posterior Cingulate Cortex
PCL-5: PTSD Checklist for DSM-5
PCS: Post-concussion Syndrome
PDEQ: Peritraumatic Dissociative Experiences Questionnaire
PE: Prolonged Exposure Therapy
PEC: Power-envelope Correlation
PFC: Prefrontal Cortex
PHQ-9: Patient Health Questionnaire
PTSD: Post-traumatic Stress Disorder
PTSD+DS: Post-traumatic Stress Disorder Dissociative Subtype
PTSD+ELS: Post-traumatic Stress Disorder with Early Life Stress
PTSD-DS: Post-traumatic Stress Disorder without Dissociative Subtype
PTSD-ELS: Post-traumatic Stress without Early Life Stress
rACC: Rostral ACC
ROI: Region of Interest
RSDI: Response to Script Driven Imaginary Scale
RSDI: State Depersonalization/Derealization Symptoms
rs-fMRI: Resting-state Functional Magnetic Resonance Imaging.
SC: Superior Collicus
SCID: Structured Clinical Interview for DSM
SCR: Skin Conductance Responding
SEC: Statistic Effective Connectivity
SFC: Statistic Functional Connectivity
sgACC: Subgenual ACC
STAI: State–trait Anxiety Inventory
SVM: Support Vector Machine
TE + EF: Trauma Exposed with Impaired Executive Functioning
TE: Trauma Exposed
TE-avgEF: Trauma Exposed with Average EF
TE-EF: Trauma-exposed without EF
TEHC: Trauma-exposed Healthy Control
TF: Trauma-focused
TMS: Transcranial Magnetic Stimulation
VAN: Ventral Attention Network
VBM: Voxel-based Morphometry
vDEC: variance of Dynamic Effective Connectivity
vDFC: variance of Dynamic Functional Connectivity
vmPFC: ventromedial PFC
WHOQOL: World Health Organization Quality-of-life Scale

## Supplementary material

The supplementary material for this article can be found at http://doi.org/10.1017/S0033291725001229.
