# Supplementary material for: PTSD subtypes and their underlying neural biomarkers: a systematic review
Source: Psychol Med. 2025 May 22;55:e153. doi: 10.1017/S0033291725001229 (PMC12115270; doi:10.1017/S0033291725001229)
Supplement: Zhang et al. supplementary material [file S0033291725001229sup001.zip › supplimentary_2024.03.24_cz_cleaned.docx]

**SUPPLEMENTARY INFORMATION**

**PTSD subtypes and their underlying neural biomarkers: A systematic review**

**Contents**

Method ………………………………...……….…..….…..….…..….…..2-4

Results………………………………...……….…..….…..….…..….…..4-22

Supplemental References ………………………………...……….…..….23-27

**Methods**

1. **Study identification and key words**

The selection of trauma-related keywords is informed by the PTSD Checklist for DSM-5 (PCL-5) (Blevins, Weathers, Davis, Witte, & Domino, 2015). The following keyword search was conducted: The following keyword search was conducted: (Posttraumatic stress disorder OR post-traumatic stress disorder OR PTSD OR trauma* OR stress* OR abuse OR violent* OR assault OR rape OR accident OR injury OR illness OR bereavement OR grief OR disaster OR hurricane OR earthquake OR combat OR war OR hostage) AND (“Biotype” OR “heterogeneity” OR “subtype” OR “heterogeneous” OR “classification” OR “clustering” OR “Biomarker” OR “Neurobiological substrate” OR “Neural Biomarker”) AND (Functional Magnetic Resonance Imaging* OR fMRI* OR resting-state* OR rs-fMRI* OR task-based* OR task-based fMRI* OR MRI OR EEG OR DTI). Studies with participants that meet sub-threshold PTSD diagnostic criteria and full PTSD diagnostic criteria were included; sub-threshold PTSD participants must have encountered one traumatic event, and PTSD diagnosis was assessed and diagnosed using either one or more of the following assessments: Structured Clinical Interview for the Diagnostic and Statistical Manual of Mental Disorders, Clinical Administered PTSD Scale (CAPS-5), and PTSD Checklist-5 (Weathers et al., 2018).

**1.1 Study screening and data extraction**

Using Covidence, the two reviewers (CZ and NP) were in complete agreement (100%) during the initial screening based on the abstract. The reviewers then independently completed the full-article review using the inclusion and exclusion criteria. Any discrepancy between the two reviewers was addressed through a discussion meeting. Initial agreement at the full article review stage was 84%, with disagreements resolved by discussion between the two reviewers. The data were collected from the full-text and entered by the two reviewers into a data extraction sheet/excel sheet. The data table was categorized by sample population (total sample size, group sample size, comorbidity, type of trauma), analysis methods (top-down vs. bottom-up data-driven approach, biotype, imaging modality, imaging features, atlases/parcellation, statistical methods, inclusion of controls in the analysis, dimensionality reduction method, cluster number selection, number of group /clusters according to biotype), biotype reproducibility strategies (internal validation, external validation, association between biotypes and clinical/behavior measures, classification among biotypes, treatment outcome prediction using biotypes if present), most discriminative features/biomarkers, description of the main findings of each paper, and relevant papers found in the selected papers.

**1.2 Quality assessment**

The quality of the papers was assessed using the Strengthening the Reporting of Observational Studies in Epidemiology (STROBE) guideline (von Elm et al., 2014). STROBE provides recommendations for the reporting of observational studies that examine the relationship between exposure factors and health outcomes. The guideline evaluates the introduction (background/rational, objectives), methods (study design, setting, participation, variables, data sources/measurement, bias, study size, quantitative variables, statistical methods), results (participants, descriptive data, outcome data, main results, other analyses), discussion (key results, limitations, interpretation, generalizability), and funding using 22 items. Using the STROBE guideline for cross-sectional studies, each article is given a score of 1 or 0 for each item; 1 meets the guideline criteria recommendation. Two reviewers (CZ and NP) independently reviewed the selected articles following each item and recorded the results in an excel sheet. Discrepancies in the article evaluation were identified as each reviewer independently reviewed each other’s evaluation record, and each reviewer then recorded the reasons for each evaluation with discrepancy, and the discrepancy was then addressed through a discussion meeting. After resolving the discrepancy, each research article was assigned a score between 0 and 22.

**Results**

**1.1 Dissociative subtype:**

Nineteen studies used the top-down approach to differentiate between PTSD with dissociative symptoms (PTSD+DS) and without dissociative symptoms (PTSD-DS). Participants are categorized based on clinical assessment using the DSM criteria, followed by the extraction of group-level neurobiological differences through structural and functional neuroimaging data (Figure 4). Biological heterogeneity within this subtype is presented through examining within-network connectivity, followed by between-network differences. Consistent results are reported if more than 2 literature reported similar direction of connectivity within and between-network.

*Between and Within network connectivity*

*Summary: Within network connectivity*

Limited research examined the within-network connectivity difference between PTSD+DS and PTSD-DS, varying from 1 to 2 studies per network. Current literature highlights greater vmPFC pattern within anterior-DMN, increased within-SN connectivity in PTSD+DS, though more research is needed due to the limited number of literature reviewed. One study reported increased connectivity of frontal pole within ECN. Two studies found contrasting rs-FC within BGN, again posing the demand of more studies.

***Within Default mode network (DMN)*** is a task-negative network characterized by multiple hubs that are active during rest, engaging in self-referential and/or self-reflective processes. Key brain regions involved in the DMN include the ventromedial prefrontal cortex (vmPFC), precuneus, and PCC. The current literature highlights specific DMN brain regions that display distinct characteristics between PTSD+DS and PTSD-DS, including the vmPFC, and the inferior temporal gyrus. Specifically, PTSD+DS participants exhibited greater resting-state activation variability in the right vmPFC (Nicholson et al., 2019; Nicholson et al., 2020)*,* measured by mean amplitude lower-frequency fluctuation (mALFF). Within-DMN volume alterations were found in PTSD+DS compared to PTSD-DS, with reduced grey matter volume (GMV) in the right inferior temporal gyrus and increased volume in hippocampal subfields and posterior cingulate gyrus (Daniels, Frewen, Theberge, & Lanius, 2016; Wolf et al., 2023).

***Within Salience network (SN)***, responsible for filtering and selecting emotionally salient stimuli, influences cognitive, emotional, and behavioral responses to these stimuli (Akiki et al., 2017). In PTSD, this network reinforces stimuli associated with PTSD-related experiences. SN brain hubs include the amygdala, insula, and ACC. The literature posits increased within-SN connectivity in PTSD+DS, compared to PTSD-DS(Nicholson et al., 2020; Nicholson et al., 2016). Studies found reduced mALFF in the left amygdala in PTSD+DS(Nicholson et al., 2019). Reduced fractional anisotropy (FA), indicating impaired white matter integrity, is observed in tracts connecting the left amygdala and hippocampus in PTSD+DS versus PTSD-DS (Sierk, Manthey, Brakemeier, Walter, & Daniels, 2021).

***Within Executive control network (ECN),*** sometimes referred to as the frontoparietal control network, plays a critical role in high-order cognitive functions such as decision-making, working memory, and planning. Key brain regions involved in the ECN include the dorsal lateral prefrontal cortex (dlPFC), posterior parietal cortex, and frontal pole. Only one study reported within-ECN alteration in the dissociative subtype. This study reported the frontal pole, a hub of ECN, as a biomarker differentiating PTSD+DS from PTSD-DS. This study also reported increased mALFF in the frontal pole in PTSD+DS, compared with PTSD-DS (Nicholson et al., 2019).

***Within Basal ganglia network (BGN)*,** including the nucleus accumbens (NAcc), Putamen, Caudate, globus pallidus and subthalamic nucleus, plays an important role in emotional processes and reward processing. Compared to PTSD-DS, PTSD+DS displayed greater rs-FC within the NAcc (Olive, Makris, Densmore, McKinnon, & Lanius, 2021), and decreased mALFF of the global pallidus, putamen, and thalamus in contrast to PTSD-DS (Nicholson et al., 2019). The orbitofrontal cortex (OFC), typically associated with the reward network and the BGN, showed greater mALFF at rest in PTSD+DS compared to PTSD-DS (Nicholson et al., 2019).

*Summary: Between network connectivity*

Overall, increased rs-FC was observed in DMN-SN, DMN-brainstem, and decreased rs-FC in ECN-brainstem. Between-network result also reveals inconsistent connectivity patterns between DMN-Cerebellar, SN-brainstem, ECN-DMN, ECN-SN, thus warranting future research to further decipher the intricate nature of the underlying mechanism.

***DMN and SN:*** SN recruits neural networks to generate adaptive responses through salient internal and/or external stimuli identification (Bressler & Menon, 2010). PDMN is involved in re-experiencing of self-referential emotional memory (Brechet, Grivaz, Gauthier, & Blanke, 2018; Cavanna & Trimble, 2006; Kearney et al., 2023), and traumatic flashback (Whalley et al., 2013). The DMN exhibits divergent connectivity patterns with regions in the SN, cerebellar network, and brainstem. Compared to PTSD-DS, PTSD+DS showed increased connectivity between the DMN hubs (the precuneus, SPL, and hippocampus) and the SN hubs (the insula and amygdala). Specifically, three studies reported increased rs-FC between the precuneus and brain regions within the SN in PTSD+DS. This subtype showed connectivity from the left precuneus to both bilateral anterior and posterior insula (Harricharan et al., 2020); increased rs-FC of pDMN-SN implicates neurobiological maladaptation underlies implicit traumatic memory and salient traumatic cue in PTSD+DS (Bressler & Menon, 2010). Another finding highlighted greater connectivity between the precuneus to the left centromedial amygdala (CMA) in PTSD+DS (Nicholson et al., 2015). Furthermore, greater rs-FC was observed from the precuneus to the overall SN, particularly the posterior SN, using independent component analysis (ICA) (Nicholson et al., 2020). Moreover, increased rs-FC was noted between the SPL and amygdala in PTSD+DS, characterized by increased connectivity between the SPL and the left basolateral amygdala (Nicholson et al., 2015). Decreased FA value within the tract connecting the left hippocampus and left amygdala was highlighted (Sierk et al., 2021), suggesting irregular fiber density and white matter within the tract. This biomarker highlights the need for modified treatment targeting the maladaptive association underlies traumatic memory and stimuli

***DMN and Brainstem:*** Three studies found increased connectivity between the DMN and brainstem hubs in the PTSD+DS vs. PTSD-DS. Greater temporoparietal junction (TPJ)- periaqueductal gray (PAG) connectivity suggests neurobiological alteration underlying impaired bodily perception and self-related states of consciousness (Harricharan et al., 2016); TPJ has been implicated in depersonalization process (Blanke & Arzy, 2005). Compared to PTSD-DS, PTSD+DS participants exhibited greater rs-FC from vmPFC to brainstem, between pedunculopontine nucleus (PPN) and innate alarm system (IAS), which comprised of vmPFC, amygdala, and midbrain (Thome et al., 2019). Similarly, increased rs-FC was found between the right TPJ and the mid-brain regions, including the right superior colliculus (Olive et al., 2018) and the ventrolateral periaqueductal gray (Harricharan et al., 2016) in PTSD+DS. The parahippocampal gyrus, while not a hub within the DMN, is often associated with the DMN. Literature suggests greater rs-FC between the parahippocampal gyrus and brainstem region (pedunculopontine nuclei) in PTSD+DS vs. PTSD-DS (Thome et al., 2019). Activation dysregulation within the parahippocampal gyrus emerges as a discriminative feature, with PTSD+DS exhibiting reduced mALFF within the parahippocampal gyrus compared to PTSD-DS (Nicholson et al., 2019).

***DMN and Cerebellar:*** The hippocampus, TPJ, and SPL, key DMN hubs, exhibit inconsistent altered connectivity with the cerebellar network in PTSD+DS compared to PTSD-DS. Specifically, greater rs-FC of the anterior hippocampus – right flocculus (Rabellino et al., 2022), SPL – cerebellar culman (Nicholson et al., 2015) and vmPFC – posterior cerebellum (Rabellino et al., 2022; Thome et al., 2019) were observed in PTSD+DS relative to PTSD-DS. Additionally, one study noted reduced rs-FC between the SPL and anterior cerebellum specifically in PTSD+DS but not in PTSD-DS (Rabellino, Densmore, Harricharan, et al., 2018). Two studies reported decreased rs-FC between TPJ and cerebellar, specifically the left flocculus and anterior cerebellum (Rabellino, Densmore, Theberge, McKinnon, & Lanius, 2018; Rabellino et al., 2022).

***SN and Brainstem:*** Inconsistent altered rs-FC patterns between the SN hubs and the brainstem are observed in PTSD+DS compared to PTSD-DS. Varied rs-FC is reported between the amygdala and pedunculopontine nucleus; decreased rs-FC between amygdala and pedunculopontine nucleus corresponds to increased heart-rate variability (HRV) in PTSD+DS (Thome et al., 2022), whereas another study reported an increased amygdala rs-FC with pedunculopontine nuclei in PTSD+DS (Thome et al., 2019). Similarly, increased rs-FC of ACC-pedunculopontine nuclei is also observed in PTSD+DS in contrast to PTSD-DS (Thome et al., 2019). Furthermore, in PTSD+DS, the amygdala exhibits top-down connectivity with the periaqueductal gray, measured using dynamic causal modeling, whereas in PTSD-DS, a bottom-up connectivity pattern is noted from the periaqueductal gray to the amygdala (Nicholson et al., 2017). Compared with PTSD-DS, PTSD+DS is characterized by decreased rs-FC from insula to periaqueductal gray in brainstem and left ventral pallidum in subcortical network (Harricharan et al., 2020).

***SN and Cerebellar:*** In one study, increased rs-FC from the amygdala to a cerebellar hub is evident in PTSD+DS compared with the PTSD-DS group. Specifically, the amygdala displays greater rs-FC with insula sub-regions and the cerebellar culman in the PTSD+DS (Nicholson et al., 2016).

***ECN and Brainstem****:* Consistent decreased connectivity in ECN-brainstem network in PTSD+DS was reported by two studies. specifically, from dorsolateral prefrontal cortex (dlPFC) to left SC and vestibular nuclei (VN) (Harricharan et al., 2017; Olive et al., 2018).

***ECN and other networks:*** Findings on the between-network rs-FC of the ECN-other networks appears to vary among studies. Compared with PTSD-DS, studies found both an increase and decrease between-network rs-FC between ECN-DMN (Lebois et al., 2022; Nicholson et al., 2020). Specifically, ECN rs-FC to DMN varies depending on the regions within DMN; increased rs-FC was observed between the ECN and left middle temporal gyrus, whereas decreased rs-FC was observed between the ECN and precuneus (Lebois et al., 2022). Lebois et al. (2022); Nicholson et al. (2020) reported increased ECN-DMN using ICA approach. Moreover, decreased rs-FC of ECN-SN was found in PTSD+DS. The decreased rs-FC of ECN-SN regions was also associated with greater dissociation (Lebois et al., 2022). However, Nicholson et al. (2020) found increased ECN (dorsal PFC)-SN in PTSD+DS. Lastly, volume of right middle frontal gyrus, part of ECN, correlates with dissociative symptoms severity (Daniels et al., 2016), reinforcing ECN as a treatment target.

***Basal ganglia network (BGN) and other networks:*** Another study demonstrated increased rs-FC of the OFC - cerebellar (Rabellino, Densmore, Theberge, et al., 2018). Moreover, one study reported increased rs-FC between the right lateral OFC and the right ECN in PTSD+DS (Nicholson et al., 2020).

*Machine learning studies*

Machine learning analyses were able to classify PTSD+DS, PTSD-DS, and controls using insula sub-region rs-FC with 80.4% balanced accuracy (Harricharan et al., 2020), using mALFF maps with 91.63% balanced accuracy (Nicholson et al., 2019), and using amygdala complex rs-FC maps with 85.00% balanced accuracy (Nicholson et al., 2019). Lastly, Nicholson et al. (2020) found that rs-FC was able to predict group classification of PTSD, PTSD+DS, and healthy individuals with 80% balanced accuracy with DMN, 74%~76% accuracy with ECN, and 70%~74% accuracy with SN.

**1.1.2 Executive function (EF) subtype**

The review of the following five studies aimed to advance our understanding of cognitive deficits and their connection to the neural underpinnings of PTSD. A study by Esterman et al. (2020) focused on examining cognitive deficits in memory and executive functioning in individuals with PTSD. While they did not find a strong association between memory impairments and the ventral attention network (VAN) signature in PTSD, they observed a link between attentional impairments and lower VAN connectivity. This finding suggests that the original subtype related to memory impairments may instead be related to attentional issues in PTSD (Esterman et al., 2020).

Extending this line of investigation, Etkin et al. (2019) identified a subset of PTSD patients characterized by abnormal rs-FC within the VAN and impaired verbal memory performance. While this phenotype was not linked to symptom variations, it did prove significant in predicting a negative response to psychotherapy(Etkin et al., 2019).

In a complementary endeavor, Jagger-Rickels et al. (2021) adopted a broader perspective by exploring the entire brain network in PTSD. They found that the severity of PTSD symptoms was associated with reduced negative coupling between the BGN and ECN, particularly in the dlPFC and amygdala hubs of dysfunction (Jagger-Rickels et al., 2021). This relationship was moderated by EF, with individuals having impaired EF showing the strongest marker of BGN-ECN dysregulation, whereas those with above-average EF did not exhibit the same dysregulation of these networks.

Building on this finding, another study by Jagger-Rickels et al. (2022) introduced a distinct cognitive subtype of PTSD. The results highlighted a specific pattern of FPCN (fronto-parietal control network) and LN (limbic network) connectivity in the impaired PTSD+EF subtype, as well as the discovery of a novel above-average PTSD+EF subtype, which exhibited reduced PTSD chronicity. Both cognitive and neural functioning were found to predict PTSD chronicity(Jagger-Rickels et al., 2022).

Y. Li et al. (2020) brought a different perspective by investigating the link between rs-fMRI data, memory function and PTSD symptoms. They found that specific brain regions, namely the left middle frontal gyrus and the left precuneus, were implicated in the brain mechanisms underlying visual working memory in relation to PTSD.

In conclusion, studies assessing **executive function subtype** highlight a shift from memory to attentional impairments, revealed a nuanced perspective by dissociating memory impairments from the ventral attention network (VAN) signature in PTSD (Esterman et al., 2020). VAN alteration previously linked to poor therapy response in PTSD, indicating its clinical relevance as predictive marker of treatment response(Etkin et al., 2019). Decoupling between limbic network (LN) and FPN is moderated by EF severity, and correlated with the chronicity of PTSD (Jagger-Rickels et al., 2022; Jagger-Rickels et al., 2021). Connectivity between ECN and DMN (middle frontal gyrus and precuneus) predicted working memory function in PTSD, suggesting visual working memory’s role in PTSD deficit (Y. Li et al., 2020).

**1.1.3 Symptoms-based subtypes**

Here we aimed to consolidate findings concerning symptom dimensions in PTSD, five out of six studies are focused on the CAPS-5 and its assessment of three symptom dimensions in PTSD: hyperarousal, avoidance, and reexperiencing. Across five studies, the three dimensions exhibit varying associations with brain activity and connectivity, predominantly within the brain hubs of the SN and DMN. One out of six studies identified distinct symptom dimensions: fear and dysphoria.

Hyperarousal, marked by heightened behavioral and emotional reactions including irritability and sensitive nerves, is linked to SN brain regions. Several studies report that greater hyperarousal symptoms are associated with increased connectivity/activity within SN areas (Grupe, Wielgosz, Davidson, & Nitschke, 2016; Tursich et al., 2015). Notably, one study identifies elevated activity within the ACC, left posterior amygdala, and anterior hippocampus cluster for severe hyperarousal (Grupe et al., 2016). Conversely, decreased within-network connectivity of the posterior insula and superior temporal gyrus is linked to hyperarousal (Tursich et al., 2015). Task-based literature indicates reduced habituation of emotional images in various regions, including the superior and inferior frontal gyrus, superior and middle temporal gyrus, vmPFC, and anterior insula among greater hyperarousal symptoms.

Re-experiencing symptoms encompass flashbacks and intrusive thoughts of the traumatic incident, and is associated with DMN across several studies. In two studies, greater re-experiencing symptoms have been found to be associated with abnormalities within the vmPFC, a hub within the DMN (Grupe et al., 2016; McCurry, Frueh, Chiu, & King-Casas, 2020). Grupe et al. (2016) identified association between severe re-experiencing and decreased vmPFC activity. A task-based study revealed positive associations between greater re-experiencing symptoms and increased habituation within a network, including the superior and inferior frontal gyrus, vmPFC, superior and middle temporal gyrus, and anterior insula. Re-experiencing symptom severity was negatively correlated with decreased ACC activity (Hopper, Frewen, van der Kolk, & Lanius, 2007), and the severity was also associated with decreased white matter integrity in the right posterior cingulum bundle (Weis, Belleau, Pedersen, Miskovich, & Larson, 2018).

Avoidance symptoms entail behaviors like avoiding stimuli related to the traumatic incident. One study reports a negative correlation between avoidance symptoms and decreased activity in SN hubs: rostral ACC and subcallosal ACC (Hopper et al., 2007).

Finally, one study established symptom dimensions based on PTSD symptom scale-interview and investigated the neural basis associated with the fear and dysphoria symptoms of PTSD (Liddell et al., 2025). Fear symptoms were negatively correlated with hyperconnectivity between the anterior dorsomedial DMN (admDMN) and the temporoparietal DMN (tpDMN), while dysphoria symptoms were positively correlated with the hyperconnectivity of admDMN and tpDMN.

In summary, symptoms-based subtypes focus on the neural basis of symptom dimensions: hyperarousal, re-experiencing, and avoidance. Direction of connectivity varies. Hyperarousal is consistently associated with SN regions (ACC, amygdala, hippocampus), while avoidance severity associates with connectivity between SN and ECN (Grupe et al., 2016; Tursich et al., 2015). Severe re-experiencing, fear and dysphoria symptoms of PTSD are associated with altered DMN hub activity (vmPFC) (Grupe et al., 2016; Liddell et al., 2025; McCurry et al., 2020).

**1.1.4 Comorbidity subtype**

Exploring the intricate relationship between PTSD and comorbid MDD (PTSD+MDD) across five studies, researchers assessed distinct network connectivity abnormalities in comparison to PTSD alone (PTSD-MDD). Focusing on brain regions involved in fear and reward processing, such as the basolateral amygdala (BLA), centromedial amygdala (CMA), and NAcc, rs-FC patterns were measured across three groups: PTSD+MDD, PTSD-MDD, and trauma-exposed healthy controls (TEHC).

Irrespective of MDD comorbidity, PTSD was consistently linked to reduced connectivity in pathways relevant to fear and reward processing. However, those with comorbid PTSD+MDD displayed broader alterations in connectivity across multiple amygdala and striatal-subcortical pathways, central to both fear and reward systems. This underscores how comorbid PTSD+MDD might lead to intricate functional connectivity changes across these critical networks (Zhu et al., 2017).

Parallel findings from Yuan et al. (2019) unveil reduced rs-FC in the PTSD+MDD group, specifically between the right BLA and the left anterior cingulate cortex, and between the BLA and the bilateral putamen/pallidum(Yuan et al., 2019). Simultaneously, heightened connectivity emerged between the CMA and the left ACC/SMA within the PTSD+MDD group. Notably, the severity of MDD symptoms inversely related to the connectivity of the right BLA with the right putamen/pallidum. van Rooij et al. (2015) contributed insights into emotional processing patterns, revealing that PTSD+MDD individuals exhibited reduced activation in the subgenual ACC across all emotional picture categories compared with PTSD-MDD. Further, Lanius et al. (2007) delved into neural activation patterns and identified distinct differences between the PTSD+MDD and PTSD-MDD groups. Meanwhile, Zilcha-Mano et al. (2020) employed a support vector machine model to pinpoint rs-FC biomarkers separating individuals with standalone PTSD from those with PTSD+MDD, highlighting the role of the basal ganglia network.

In summary, studies on **comorbidity subtype** reveal consistent findings on the relationship between PTSD+MDD with a focus on brain networks involved in fear and reward processing. Comorbid PTSD+MDD revealed broader alterations in connectivity across multiple amygdala and striatal-subcortical pathways, central to both fear and reward systems (van Rooij et al., 2015; Yuan et al., 2019).

**1.1.5 Psychotherapy response subtype**

Efforts to enhance the efficacy of trauma-focused psychotherapies for PTSD have spurred studies exploring neural mechanisms and potential biomarkers of **psychotherapy response subtype**, highlighting the role of network activity in differentiating treatment responders from non-responders. Distinct resting-state network (RSN) centered on the ECN hub differentiates responders from non-responders (Zhutovsky et al., 2019). Heightened ECN and SN hub activation prior to treatment differentiates remitters and non-remitters(van Rooij, Kennis, Vink, & Geuze, 2016; van Rooij et al., 2015), which contrasts with reduced activation within DMN regions during inhibition tasks (Bryant et al., 2021).

In an effort to improve the effectiveness of trauma-focused psychotherapies (TF-CBT and EMDR) for PTSD, four studies have illuminated the intricate neural underpinnings and biomarkers associated with treatment responsiveness. Zhutovsky et al. (2019) explored if MRI/rs-fMRI data could distinguish treatment responders from non-responders both at the group and individual levels. A distinct RSN, centered on the bilateral superior frontal gyrus, exhibited differential activity between the groups. By employing machine learning analysis to make predictions at the individual level, another RSN, centered on the pre-supplementary motor area, demonstrated remarkable accuracy in distinguishing between responders and non-responders. No significant differences in gray matter volume were observed on either level. In a parallel endeavor, (van Rooij et al., 2016) investigated the persistence of PTSD symptoms post trauma-focused therapy. Patients showing persistence of symptoms exhibited heightened dACC and insula responses to negative images both before and after treatment, compared with patients showing remission and controls (van Rooij et al., 2016). Increased amygdala activation to negative images was evident before treatment. Notably, higher SN activation (dACC, insula, and amygdala) before treatment was predictive of symptom persistence, emphasizing a potential neural marker for poor PTSD treatment response (van Rooij et al., 2015).

Bryant et al. (2021) used fMRI and event-related potential (ERP) during a response inhibition task to assess inhibitory functions in PTSD patients who underwent TF-CBT. While neural responses during the task did not predict overall symptom change, reduced activation in the left precuneus and the right superior parietal cortex predicted better reduction in dysphoric symptoms. ERP responses also indicated that lower P3 peak latency patterns predicted dysphoric symptom improvement. These findings suggest that neural activity related to response inhibition can serve as a predictive biomarker for TF-CBT response in managing PTSD symptoms.

Korgaonkar et al. (2021) pursued the role of white matter brain structures in TF-CBT response in PTSD patients as compared with healthy controls. Greater reduction in dysphoric symptoms correlated with lower fractional anisotropy (FA) in white matter regions linked to limbic, frontal, thalamic, corpus callosum, and brainstem tracts. No association was found for overall or fear symptoms with white matter anisotropy. FA in the significant clusters was similar between PTSD and controls. These findings highlight white matter’s role in TF-CBT response and emphasize the need to assess specific symptom phenotypes in PTSD treatment studies.

In summary, these findings underscore the potential of distinct resting-state networks, heightened salience network activation, neural activity related to response inhibition, and white matter structures as predictors of treatment responsiveness. These findings not only enhance our understanding of treatment outcomes but also emphasize the importance of personalized approaches that consider specific symptom phenotypes in the management of PTSD.

**1.1.6 Suicidality subtype**

Two studies have delved into the intricate neural underpinnings of suicidal ideation (SI) within the context of PTSD among combat veterans. (Bomyea, Stout, & Simmons, 2019) compared fMRI neural activation of combat veterans with PTSD, with and without current SI, while performing a working memory task. Participants with SI showed reduced neural activation in key brain regions involved in cognitive control, particularly in response to interference demands during the task, implicating prefrontal brain regions in cognitive regulation as relevant to SI and temporal functioning involvement. In another study, Stumps et al. (2021) tested rs-fMRI connectivity in post-9/11 veterans with a suicide attempt history, comparing them with a psychiatric control group and a trauma control group. Employing graph-analytic and seed-based methods, the study found that the right amygdala and right middle temporal gyrus (MTG) could serve as unique neural markers of suicide ideation, characterized by abnormal connectivity across the ECN, DMN, and visual networks.

Consistently across two studies, hubs within ECN (prefrontal brain regions) and SN (right amygdala and right middle temporal gyrus) emerge as pivotal in regulating cognitive processes related to suicidality in PTSD (Bomyea et al., 2019; Stumps et al., 2021).

**1.1.7 Complex-PTSD (cPTSD) subtype**

The Complex-PTSD (cPTSD) subtype has been investigated through two notable studies. (Bryant, Tran, Williamson, & Korgaonkar, 2022) employed fMRI and EEG paradigms to explore inhibitory control neural networks in participants with cPTSD, PTSD, and healthy controls. During separate fMRI and EEG sessions, participants engaged in a response inhibition task, specifically focusing on NoGo trials. Results indicated reduced bilateral thalamic activation in cPTSD compared with both PTSD and control groups during inhibition trials, with no activation differences observed between PTSD and controls in this region. Functional connectivity between the thalamus and other cognitive control-related regions showed no group differences. The findings provide initial evidence of impaired inhibitory control neural functioning, particularly involving the thalamus, in individuals with cPTSD. These findings suggest that cPTSD differs from PTSD due to disruptions in neural processes related to response inhibition.

Similarly, Herzog et al. (2019) found that cPTSD patients showed greater difficulty in a Stroop task involving trauma-related words, with slower reaction times and more errors compared with trauma-exposed healthy controls and non-trauma-exposed healthy controls (Herzog et al., 2019). The brain regions related to cognitive control were more active in cPTSD patients when processing trauma-related words, possibly indicating a compensation mechanism for emotional distraction caused by the trauma. This suggests that cognitive control processes might play a role in managing emotional responses in cPTSD.

In summary, two task-based studies offer insight into **C-PTSD subtype.** Bryant et al. (2022) emphasize disrupted thalamus response related to response inhibition as a differentiating factor for cPTSD, while Herzog et al. (2019) highlight the role of ECN in emotional regulation within this subtype. These findings contribute to our understanding of cPTSD's neural underpinnings, potentially guiding future diagnostic and therapeutic approaches. This suggests disintegrated network connectivity within c-PTSD subtype.

**1.1.8 Early life stress subtype**

Using evolving partitions to improve classification (EPIC), Salminen et al. (2019) examined how combinations of cortical thickness, surface area, and subcortical brain volumes, alongside factors like intracranial volume, age, sex, adult trauma, and depression, could aid in distinguishing PTSD patients from controls, and how EPIC could classify ELS within both the PTSD and control groups. One consistent feature that stood out was the surface area in the right posterior cingulate (DMN hub), indicating its importance in differentiating PTSD+ELS and PTSD-ELS.

**1.1.9 Disinhibition subtype**

One study aimed to investigate impulse control deficits in individuals with PTSD by examining brain structure and connectivity changes associated with response inhibition failures and PTSD severity (Sadeh et al., 2015). Among veterans, reduced cortical thickness in specific regions linked to decision-making and emotional regulation (the right inferior and middle frontal gyri, frontal pole, left medial OFC, rostral ACC, and superior frontal gyrus) was associated with impaired response inhibition, particularly with higher PTSD symptoms (Sadeh et al., 2015). Rs-fMRI analysis revealed disrupted connections between frontal regions and networks involved in attention, memory, and response preparation.

**1.1.10 Self-blame subtype**

One study used structural and rs-fMRI to investigate the neural mechanisms underlying the connection between self-blame and PTSD symptoms in sexually assaulted women compared with non-exposed controls (Berman, Assaf, Tarrasch, & Joel, 2018). Among the sexually assaulted participants, those who expressed self-blame for the assault showed reduced GMV in the lingual gyrus and adjacent intracalcarine cortex (visual network hubs). This brain region was also influenced by intrusion symptoms and negative social reactions. Rs-FC between this brain cluster and the left anterior temporal fusiform cortex differed between sexually assaulted participants with and without PTSD. Significant association between structural and connectivity analysis and intrusion symptoms and peritraumatic dissociation suggest altered experience of visual memory may be a biomarker for self-blame.

**2.2 Data-driven approaches**

Nine studies used bottom-up data-driven approaches. Brain-based, bottom-up data-driven approaches parse heterogeneity based on neurobiological measures such as neuroimaging data, including structural MRI, resting-state fMRI data, and task-based fMRI. Zilcha-Mano et al. (2022) identified two clusters of PTSD biotypes using the structural MRI data such as volumes, surface area, and cortical thickness within the areas of interest. One cluster, referred to as the resilience cluster, was characterized by higher volume in brain regions implicated in trauma exposure, such as the thalamus and rostral middle frontal gyrus.

Five studies identified brain biotypes based on resting-state fMRI data. Strigo, Spadoni, and Simmons (2022) generated two PTSD biotypes using resting-state fMRI data from 57 participants. Maron-Katz et al. (2020) identified two subgroups within PTSD using rsFC data. Subgroups differed clinically on levels of re-experiencing symptoms and direction of connectivity between visual and sensorimotor network. Stout, Harle, Norman, Simmons, and Spadoni (2021) identified a biotype characterized by lower insular to inferior parietal cortex (IPC) connectivity, higher pregenual ACC (pgACC) to posterior midcingulate cortex, and pgACC-IPC. This biotype was associated with improvement in PTSD symptoms from integrated-prolonged exposure therapy (Stout et al., 2021). Another study used both dynamic and static rs-fMRI data, and generated 2 to 3 clusters depending on the feature selection methods (sequential feature ranking [SFR], sequential feature selection [SFS], genetic algorithms[GA]) and clustering methods (density peak clustering[DPC], ordering points to identify the clustering structure [OPTICS], hierarchical clustering) that were used; the following combinations achieved similarity of 100% with clinical symptoms: SFS + OPTICS, GA + DPC, and GA+ OPTICS (Zhao et al., 2018). GA + OPTICS combination used features located in DMN, BGN, and semantic cognition and attention network (SCAN).

Stevens et al. (2021) generated four clusters of PTSD subtypes using the fMRI data collected from participants that engaged in a variety of task-based paradigms such as an inhibition task, stop-signal task, and reward task, using hierarchical agglomerated clustering (Stevens et al., 2021). Moreover, Ahrenholtz et al. (2021) identified three biotypes using fear conditioning and extinction fMRI tasks. These three biotypes were differentiated by within the SN, DMN, and visual processing network.

In addition to resting-state fMRI data, two studies utilized data-driven approaches using resting-state EEG data. Zhang et al. (2021) identified 2 PTSD subtypes differentiated by biomarker called power-envelop-based connectivity (PEC); a connectivity measure based on the correlation of EEG's spontaneous oscillatory activity. Using sparse k-means clustering, the two PTSD+MDD subtypes exhibits different PEC directionality within frontoparietal control network and DMN respectively (Zhang et al., 2021). In addition to PEC, Q. Li et al. (2022) used Granger causality (GC) and phase lag index (wPLI) data to generate two PTSD subtypes. The two subtypes differ within parietal, temporal, visual areas, and posterior cingulate cortex connectivity, with one subtype reporting greater resemblance with the control population.

**Reference:**

Ahrenholtz, R., Hiser, J., Ross, M. C., Privratsky, A., Sartin-Tarm, A., James, G. A., & Cisler, J. M. (2021). Unique neurocircuitry activation profiles during fear conditioning and extinction among women with posttraumatic stress disorder. *J Psychiatr Res, 141*, 257-266. doi:10.1016/j.jpsychires.2021.07.007

Akiki, T. J., Averill, C. L., Wrocklage, K. M., Schweinsburg, B., Scott, J. C., Martini, B., . . . Abdallah, C. G. (2017). The Association of PTSD Symptom Severity with Localized Hippocampus and Amygdala Abnormalities. *Chronic Stress (Thousand Oaks), 1*. doi:10.1177/2470547017724069

Berman, Z., Assaf, Y., Tarrasch, R., & Joel, D. (2018). Assault-related self-blame and its association with PTSD in sexually assaulted women: an MRI inquiry. *Soc Cogn Affect Neurosci, 13*(7), 775-784. doi:10.1093/scan/nsy044

Blanke, O., & Arzy, S. (2005). The out-of-body experience: disturbed self-processing at the temporo-parietal junction. *Neuroscientist, 11*(1), 16-24. doi:10.1177/1073858404270885

Blevins, C. A., Weathers, F. W., Davis, M. T., Witte, T. K., & Domino, J. L. (2015). The Posttraumatic Stress Disorder Checklist for DSM-5 (PCL-5): Development and Initial Psychometric Evaluation. *J Trauma Stress, 28*(6), 489-498. doi:10.1002/jts.22059

Bomyea, J., Stout, D. M., & Simmons, A. N. (2019). Attenuated prefrontal and temporal neural activity during working memory as a potential biomarker of suicidal ideation in veterans with PTSD. *J Affect Disord, 257*, 607-614. doi:10.1016/j.jad.2019.07.050

Brechet, L., Grivaz, P., Gauthier, B., & Blanke, O. (2018). Common Recruitment of Angular Gyrus in Episodic Autobiographical Memory and Bodily Self-Consciousness. *Front Behav Neurosci, 12*, 270. doi:10.3389/fnbeh.2018.00270

Bressler, S. L., & Menon, V. (2010). Large-scale brain networks in cognition: emerging methods and principles. *Trends Cogn Sci, 14*(6), 277-290. doi:10.1016/j.tics.2010.04.004

Bryant, R. A., Tran, J., Williamson, T., & Korgaonkar, M. S. (2022). Neural processes during response inhibition in complex posttraumatic stress disorder. *Depress Anxiety, 39*(4), 307-314. doi:10.1002/da.23235

Bryant, R. A., Williamson, T., Erlinger, M., Felmingham, K. L., Malhi, G., Hinton, M., . . . Korgaonkar, M. S. (2021). Neural activity during response inhibition associated with improvement of dysphoric symptoms of PTSD after trauma-focused psychotherapy-an EEG-fMRI study. *Transl Psychiatry, 11*(1), 218. doi:10.1038/s41398-021-01340-8

Cavanna, A. E., & Trimble, M. R. (2006). The precuneus: a review of its functional anatomy and behavioural correlates. *Brain, 129*(Pt 3), 564-583. doi:10.1093/brain/awl004

Daniels, J. K., Frewen, P., Theberge, J., & Lanius, R. A. (2016). Structural brain aberrations associated with the dissociative subtype of post-traumatic stress disorder. *Acta Psychiatr Scand, 133*(3), 232-240. doi:10.1111/acps.12464

Esterman, M., Stumps, A., Jagger-Rickels, A., Rothlein, D., DeGutis, J., Fortenbaugh, F., . . . McGlinchey, R. (2020). Evaluating the evidence for a neuroimaging subtype of posttraumatic stress disorder. *Sci Transl Med, 12*(568). doi:10.1126/scitranslmed.aaz9343

Etkin, A., Maron-Katz, A., Wu, W., Fonzo, G. A., Huemer, J., Vertes, P. E., . . . O'Hara, R. (2019). Using fMRI connectivity to define a treatment-resistant form of post-traumatic stress disorder. *Sci Transl Med, 11*(486). doi:10.1126/scitranslmed.aal3236

Grupe, D. W., Wielgosz, J., Davidson, R. J., & Nitschke, J. B. (2016). Neurobiological correlates of distinct post-traumatic stress disorder symptom profiles during threat anticipation in combat veterans. *Psychol Med, 46*(9), 1885-1895. doi:10.1017/S0033291716000374

Harricharan, S., Nicholson, A. A., Densmore, M., Theberge, J., McKinnon, M. C., Neufeld, R. W. J., & Lanius, R. A. (2017). Sensory overload and imbalance: Resting-state vestibular connectivity in PTSD and its dissociative subtype. *Neuropsychologia, 106*, 169-178. doi:10.1016/j.neuropsychologia.2017.09.010

Harricharan, S., Nicholson, A. A., Thome, J., Densmore, M., McKinnon, M. C., Theberge, J., . . . Lanius, R. A. (2020). PTSD and its dissociative subtype through the lens of the insula: Anterior and posterior insula resting-state functional connectivity and its predictive validity using machine learning. *Psychophysiology, 57*(1), e13472. doi:10.1111/psyp.13472

Harricharan, S., Rabellino, D., Frewen, P. A., Densmore, M., Theberge, J., McKinnon, M. C., . . . Lanius, R. A. (2016). fMRI functional connectivity of the periaqueductal gray in PTSD and its dissociative subtype. *Brain Behav, 6*(12), e00579. doi:10.1002/brb3.579

Herzog, J. I., Niedtfeld, I., Rausch, S., Thome, J., Mueller-Engelmann, M., Steil, R., . . . Schmahl, C. (2019). Increased recruitment of cognitive control in the presence of traumatic stimuli in complex PTSD. *Eur Arch Psychiatry Clin Neurosci, 269*(2), 147-159. doi:10.1007/s00406-017-0822-x

Hopper, J. W., Frewen, P. A., van der Kolk, B. A., & Lanius, R. A. (2007). Neural correlates of reexperiencing, avoidance, and dissociation in PTSD: symptom dimensions and emotion dysregulation in responses to script-driven trauma imagery. *J Trauma Stress, 20*(5), 713-725. doi:10.1002/jts.20284

Jagger-Rickels, A., Rothlein, D., Stumps, A., Evans, T. C., Bernstein, J., Milberg, W., . . . Esterman, M. (2022). An executive function subtype of PTSD with unique neural markers and clinical trajectories. *Transl Psychiatry, 12*(1), 262. doi:10.1038/s41398-022-02011-y

Jagger-Rickels, A., Stumps, A., Rothlein, D., Park, H., Fortenbaugh, F., Zuberer, A., . . . Esterman, M. (2021). Impaired executive function exacerbates neural markers of posttraumatic stress disorder. *Psychol Med*, 1-14. doi:10.1017/S0033291721000842

Kearney, B. E., Terpou, B. A., Densmore, M., Shaw, S. B., Theberge, J., Jetly, R., . . . Lanius, R. A. (2023). How the body remembers: Examining the default mode and sensorimotor networks during moral injury autobiographical memory retrieval in PTSD. *Neuroimage Clin, 38*, 103426. doi:10.1016/j.nicl.2023.103426

Korgaonkar, M. S., Felmingham, K. L., Klimova, A., Erlinger, M., Williams, L. M., & Bryant, R. A. (2021). White matter anisotropy and response to cognitive behavior therapy for posttraumatic stress disorder. *Transl Psychiatry, 11*(1), 14. doi:10.1038/s41398-020-01143-3

Lanius, R. A., Frewen, P. A., Girotti, M., Neufeld, R. W., Stevens, T. K., & Densmore, M. (2007). Neural correlates of trauma script-imagery in posttraumatic stress disorder with and without comorbid major depression: a functional MRI investigation. *Psychiatry Res, 155*(1), 45-56. doi:10.1016/j.pscychresns.2006.11.006

Lebois, L. A. M., Kumar, P., Palermo, C. A., Lambros, A. M., O'Connor, L., Wolff, J. D., . . . Kaufman, M. L. (2022). Deconstructing dissociation: a triple network model of trauma-related dissociation and its subtypes. *Neuropsychopharmacology, 47*(13), 2261-2270. doi:10.1038/s41386-022-01468-1

Li, Q., Coulson Theodorsen, M., Konvalinka, I., Eskelund, K., Karstoft, K. I., Bo Andersen, S., & Andersen, T. S. (2022). Resting-state EEG functional connectivity predicts post-traumatic stress disorder subtypes in veterans. *J Neural Eng, 19*(6). doi:10.1088/1741-2552/ac9aaf

Li, Y., Zhu, H., Ren, Z., Lui, S., Yuan, M., Gong, Q., . . . Zhang, W. (2020). Exploring memory function in earthquake trauma survivors with resting-state fMRI and machine learning. *BMC Psychiatry, 20*(1), 43. doi:10.1186/s12888-020-2452-5

Liddell, B. J., Das, P., Malhi, G. S., Felmingham, K. L., Outhred, T., Cheung, J., . . . Bryant, R. A. (2025). Opponent intrinsic brain network connectivity profiles associated with posttraumatic fear and dysphoria symptoms in trauma-exposed refugees. *Psychol Trauma, 17*(1), 77-87. doi:10.1037/tra0001608

Maron-Katz, A., Zhang, Y., Narayan, M., Wu, W., Toll, R. T., Naparstek, S., . . . Etkin, A. (2020). Individual Patterns of Abnormality in Resting-State Functional Connectivity Reveal Two Data-Driven PTSD Subgroups. *Am J Psychiatry, 177*(3), 244-253. doi:10.1176/appi.ajp.2019.19010060

McCurry, K. L., Frueh, B. C., Chiu, P. H., & King-Casas, B. (2020). Opponent Effects of Hyperarousal and Re-experiencing on Affective Habituation in Posttraumatic Stress Disorder. *Biol Psychiatry Cogn Neurosci Neuroimaging, 5*(2), 203-212. doi:10.1016/j.bpsc.2019.09.006

Nicholson, A. A., Densmore, M., Frewen, P. A., Theberge, J., Neufeld, R. W., McKinnon, M. C., & Lanius, R. A. (2015). The Dissociative Subtype of Posttraumatic Stress Disorder: Unique Resting-State Functional Connectivity of Basolateral and Centromedial Amygdala Complexes. *Neuropsychopharmacology, 40*(10), 2317-2326. doi:10.1038/npp.2015.79

Nicholson, A. A., Densmore, M., McKinnon, M. C., Neufeld, R. W. J., Frewen, P. A., Theberge, J., . . . Lanius, R. A. (2019). Machine learning multivariate pattern analysis predicts classification of posttraumatic stress disorder and its dissociative subtype: a multimodal neuroimaging approach. *Psychol Med, 49*(12), 2049-2059. doi:10.1017/S0033291718002866

Nicholson, A. A., Friston, K. J., Zeidman, P., Harricharan, S., McKinnon, M. C., Densmore, M., . . . Lanius, R. A. (2017). Dynamic causal modeling in PTSD and its dissociative subtype: Bottom-up versus top-down processing within fear and emotion regulation circuitry. *Hum Brain Mapp, 38*(11), 5551-5561. doi:10.1002/hbm.23748

Nicholson, A. A., Harricharan, S., Densmore, M., Neufeld, R. W. J., Ros, T., McKinnon, M. C., . . . Lanius, R. A. (2020). Classifying heterogeneous presentations of PTSD via the default mode, central executive, and salience networks with machine learning. *Neuroimage Clin, 27*, 102262. doi:10.1016/j.nicl.2020.102262

Nicholson, A. A., Sapru, I., Densmore, M., Frewen, P. A., Neufeld, R. W., Theberge, J., . . . Lanius, R. A. (2016). Unique insula subregion resting-state functional connectivity with amygdala complexes in posttraumatic stress disorder and its dissociative subtype. *Psychiatry Res Neuroimaging, 250*, 61-72. doi:10.1016/j.pscychresns.2016.02.002

Olive, I., Densmore, M., Harricharan, S., Theberge, J., McKinnon, M. C., & Lanius, R. (2018). Superior colliculus resting state networks in post-traumatic stress disorder and its dissociative subtype. *Hum Brain Mapp, 39*(1), 563-574. doi:10.1002/hbm.23865

Olive, I., Makris, N., Densmore, M., McKinnon, M. C., & Lanius, R. A. (2021). Altered basal forebrain BOLD signal variability at rest in posttraumatic stress disorder: A potential candidate vulnerability mechanism for neurodegeneration in PTSD. *Hum Brain Mapp, 42*(11), 3561-3575. doi:10.1002/hbm.25454

Rabellino, D., Densmore, M., Harricharan, S., Jean, T., McKinnon, M. C., & Lanius, R. A. (2018). Resting-state functional connectivity of the bed nucleus of the stria terminalis in post-traumatic stress disorder and its dissociative subtype. *Hum Brain Mapp, 39*(3), 1367-1379. doi:10.1002/hbm.23925

Rabellino, D., Densmore, M., Theberge, J., McKinnon, M. C., & Lanius, R. A. (2018). The cerebellum after trauma: Resting-state functional connectivity of the cerebellum in posttraumatic stress disorder and its dissociative subtype. *Hum Brain Mapp, 39*(8), 3354-3374. doi:10.1002/hbm.24081

Rabellino, D., Thome, J., Densmore, M., Theberge, J., McKinnon, M. C., & Lanius, R. A. (2022). The Vestibulocerebellum and the Shattered Self: a Resting-State Functional Connectivity Study in Posttraumatic Stress Disorder and Its Dissociative Subtype. *Cerebellum*. doi:10.1007/s12311-022-01467-4

Sadeh, N., Spielberg, J. M., Miller, M. W., Milberg, W. P., Salat, D. H., Amick, M. M., . . . McGlinchey, R. E. (2015). Neurobiological indicators of disinhibition in posttraumatic stress disorder. *Hum Brain Mapp, 36*(8), 3076-3086. doi:10.1002/hbm.22829

Salminen, L. E., Morey, R. A., Riedel, B. C., Jahanshad, N., Dennis, E. L., & Thompson, P. M. (2019). Adaptive Identification of Cortical and Subcortical Imaging Markers of Early Life Stress and Posttraumatic Stress Disorder. *J Neuroimaging, 29*(3), 335-343. doi:10.1111/jon.12600

Sierk, A., Manthey, A., Brakemeier, E. L., Walter, H., & Daniels, J. K. (2021). The dissociative subtype of posttraumatic stress disorder is associated with subcortical white matter network alterations. *Brain Imaging Behav, 15*(2), 643-655. doi:10.1007/s11682-020-00274-x

Stevens, J. S., Harnett, N. G., Lebois, L. A. M., van Rooij, S. J. H., Ely, T. D., Roeckner, A., . . . Ressler, K. J. (2021). Brain-Based Biotypes of Psychiatric Vulnerability in the Acute Aftermath of Trauma. *Am J Psychiatry, 178*(11), 1037-1049. doi:10.1176/appi.ajp.2021.20101526

Stout, D. M., Harle, K. M., Norman, S. B., Simmons, A. N., & Spadoni, A. D. (2021). Resting-state connectivity subtype of comorbid PTSD and alcohol use disorder moderates improvement from integrated prolonged exposure therapy in Veterans. *Psychol Med*, 1-10. doi:10.1017/S0033291721001513

Strigo, I. A., Spadoni, A. D., & Simmons, A. N. (2022). Understanding Pain and Trauma Symptoms in Veterans From Resting-State Connectivity: Unsupervised Modeling. *Front Pain Res (Lausanne), 3*, 871961. doi:10.3389/fpain.2022.871961

Stumps, A., Jagger-Rickels, A., Rothlein, D., Amick, M., Park, H., Evans, T., . . . Esterman, M. (2021). Connectome-based functional connectivity markers of suicide attempt. *J Affect Disord, 283*, 430-440. doi:10.1016/j.jad.2020.11.061

Thome, J., Densmore, M., Koppe, G., Terpou, B., Theberge, J., McKinnon, M. C., & Lanius, R. A. (2019). Back to the Basics: Resting State Functional Connectivity of the Reticular Activation System in PTSD and its Dissociative Subtype. *Chronic Stress (Thousand Oaks), 3*, 2470547019873663. doi:10.1177/2470547019873663

Thome, J., Densmore, M., Terpou, B. A., Theberge, J., McKinnon, M. C., & Lanius, R. A. (2022). Contrasting Associations Between Heart Rate Variability and Brainstem-Limbic Connectivity in Posttraumatic Stress Disorder and Its Dissociative Subtype: A Pilot Study. *Front Behav Neurosci, 16*, 862192. doi:10.3389/fnbeh.2022.862192

Tursich, M., Ros, T., Frewen, P. A., Kluetsch, R. C., Calhoun, V. D., & Lanius, R. A. (2015). Distinct intrinsic network connectivity patterns of post-traumatic stress disorder symptom clusters. *Acta Psychiatr Scand, 132*(1), 29-38. doi:10.1111/acps.12387

van Rooij, S. J., Kennis, M., Vink, M., & Geuze, E. (2016). Predicting Treatment Outcome in PTSD: A Longitudinal Functional MRI Study on Trauma-Unrelated Emotional Processing. *Neuropsychopharmacology, 41*(4), 1156-1165. doi:10.1038/npp.2015.257

van Rooij, S. J., Rademaker, A. R., Kennis, M., Vink, M., Kahn, R. S., & Geuze, E. (2015). Neural correlates of trauma-unrelated emotional processing in war veterans with PTSD. *Psychol Med, 45*(3), 575-587. doi:10.1017/S0033291714001706

von Elm, E., Altman, D. G., Egger, M., Pocock, S. J., Gotzsche, P. C., Vandenbroucke, J. P., & Initiative, S. (2014). The Strengthening the Reporting of Observational Studies in Epidemiology (STROBE) Statement: guidelines for reporting observational studies. *Int J Surg, 12*(12), 1495-1499. doi:10.1016/j.ijsu.2014.07.013

Weathers, F. W., Bovin, M. J., Lee, D. J., Sloan, D. M., Schnurr, P. P., Kaloupek, D. G., . . . Marx, B. P. (2018). The Clinician-Administered PTSD Scale for DSM-5 (CAPS-5): Development and initial psychometric evaluation in military veterans. *Psychol Assess, 30*(3), 383-395. doi:10.1037/pas0000486

Weis, C. N., Belleau, E. L., Pedersen, W. S., Miskovich, T. A., & Larson, C. L. (2018). Structural Connectivity of the Posterior Cingulum Is Related to Reexperiencing Symptoms in Posttraumatic Stress Disorder. *Chronic Stress (Thousand Oaks), 2*. doi:10.1177/2470547018807134

Whalley, M. G., Kroes, M. C., Huntley, Z., Rugg, M. D., Davis, S. W., & Brewin, C. R. (2013). An fMRI investigation of posttraumatic flashbacks. *Brain Cogn, 81*(1), 151-159. doi:10.1016/j.bandc.2012.10.002

Wolf, E. J., Hawn, S. E., Sullivan, D. R., Miller, M. W., Sanborn, V., Brown, E., . . . Milberg, W. P. (2023). Neurobiological and genetic correlates of the dissociative subtype of posttraumatic stress disorder. *J Psychopathol Clin Sci, 132*(4), 409-427. doi:10.1037/abn0000795

Yuan, M., Pantazatos, S. P., Zhu, H., Li, Y., Miller, J. M., Rubin-Falcone, H., . . . John Mann, J. (2019). Altered amygdala subregion-related circuits in treatment-naive post-traumatic stress disorder comorbid with major depressive disorder. *Eur Neuropsychopharmacol, 29*(10), 1092-1101. doi:10.1016/j.euroneuro.2019.07.238

Zhang, Y., Wu, W., Toll, R. T., Naparstek, S., Maron-Katz, A., Watts, M., . . . Etkin, A. (2021). Identification of psychiatric disorder subtypes from functional connectivity patterns in resting-state electroencephalography. *Nat Biomed Eng, 5*(4), 309-323. doi:10.1038/s41551-020-00614-8

Zhao, X., Rangaprakash, D., Yuan, B., Denney, T. S., Jr., Katz, J. S., Dretsch, M. N., & Deshpande, G. (2018). Investigating the Correspondence of Clinical Diagnostic Grouping With Underlying Neurobiological and Phenotypic Clusters Using Unsupervised Machine Learning. *Front Appl Math Stat, 4*. doi:10.3389/fams.2018.00025

Zhu, X., Helpman, L., Papini, S., Schneier, F., Markowitz, J. C., Van Meter, P. E., . . . Neria, Y. (2017). Altered resting state functional connectivity of fear and reward circuitry in comorbid PTSD and major depression. *Depress Anxiety, 34*(7), 641-650. doi:10.1002/da.22594

Zhutovsky, P., Thomas, R. M., Olff, M., van Rooij, S. J. H., Kennis, M., van Wingen, G. A., & Geuze, E. (2019). Individual prediction of psychotherapy outcome in posttraumatic stress disorder using neuroimaging data. *Transl Psychiatry, 9*(1), 326. doi:10.1038/s41398-019-0663-7

Zilcha-Mano, S., Zhu, X., Lazarov, A., Suarez-Jimenez, B., Helpman, L., Kim, Y., . . . Rutherford, B. R. (2022). Structural brain features signaling trauma, PTSD, or resilience? A systematic exploration. *Depress Anxiety, 39*(10-11), 695-705. doi:10.1002/da.23275

Zilcha-Mano, S., Zhu, X., Suarez-Jimenez, B., Pickover, A., Tal, S., Such, S., . . . Rutherford, B. R. (2020). Diagnostic and Predictive Neuroimaging Biomarkers for Posttraumatic Stress Disorder. *Biol Psychiatry Cogn Neurosci Neuroimaging, 5*(7), 688-696. doi:10.1016/j.bpsc.2020.03.010
