# Supplementary material for: PTSD subtypes and their underlying neural biomarkers: a systematic review
Source: Psychol Med. 2025 May 22;55:e153. doi: 10.1017/S0033291725001229 (PMC12115270; doi:10.1017/S0033291725001229)
Supplement: Zhang et al. supplementary material [file S0033291725001229sup001.zip › Figures_03_24_25.docx]

Figure 1: This figure illustrates the two approaches in understanding the subtypes of PTSD, including DSM-based top-down method, symptom-based group analysis, and brain-based data-driven method. The arrow indicates the order of each step in generating subtypes. The orange triangles illustrates the steps to generate PTSD subtypes using symptom-based group analysis approach. The green triangle on the right describes the steps in creating subtypes using brain-based data-driven approach.


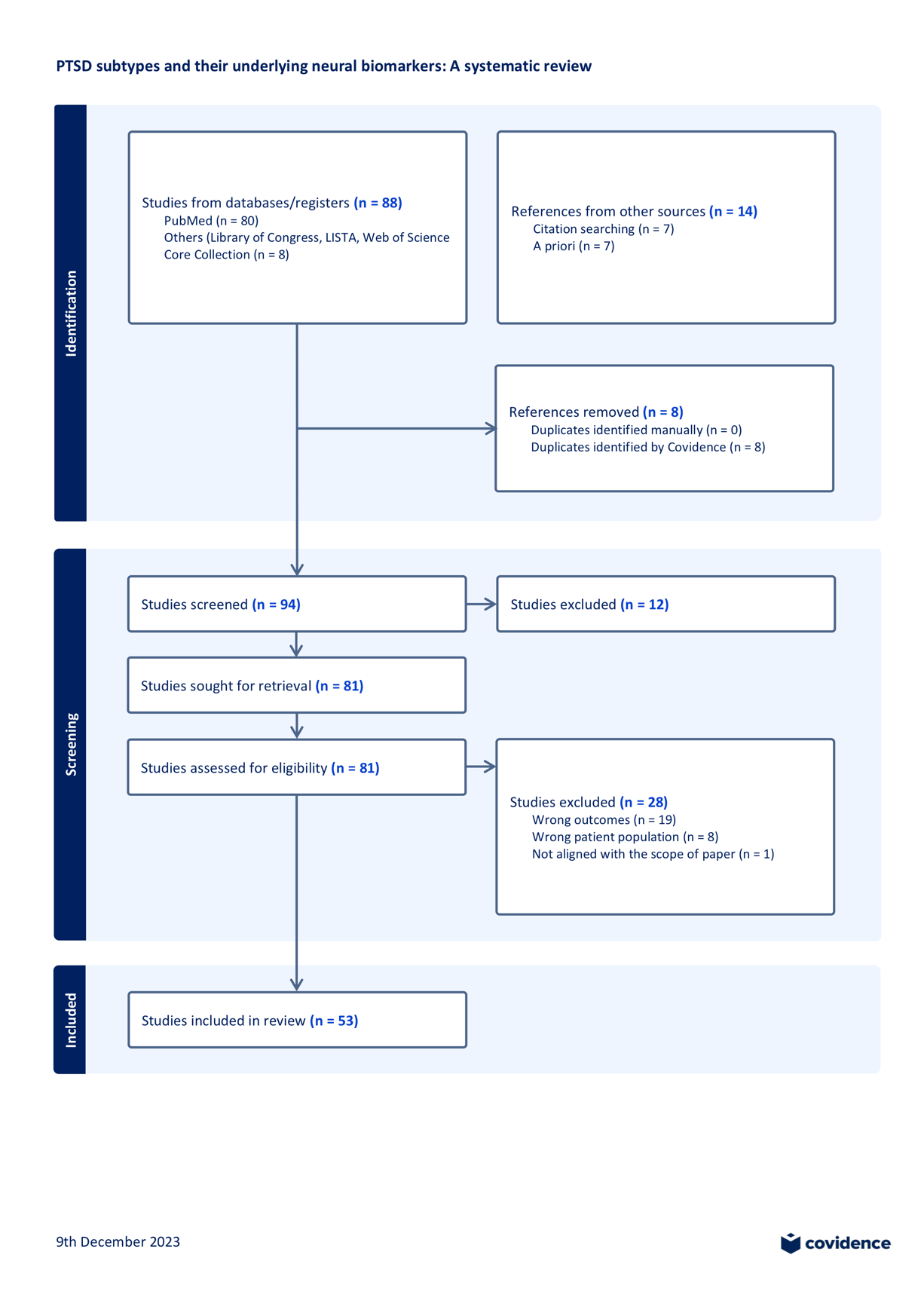


Figure 2: Flowchart for study inclusion. Initial literature search was conducted via PubMed, Library of Congress, LISTA, Web of Science Core Collection using key words (refer to supplementary material methods). Additional literature was added based on reviewing reference lists and a priori knowledge. The PRISMA review methods were used to evaluate the research articles. A final number of 53 studies were included. For additional details, refer to supplementary.

Figure 3: This figure illustrates the study overview through the following four figures. Figure 3A) This graph presents the percentage of available studies using each of the two identified approaches: symptom-based group analysis and brain-based data driven approach Figure 3B) This graph summarizes the percentage of selected studies that investigated different categories of PTSD subtypes using a top-down approaches; ten subtypes of PTSD have been under investigation, with dissociative subtype being the most researched subtype as 40% of studies investigated dissociative subtypes. Figure 3C) This graph illustrates the percentage of studies that utilized each type of neuroimaging modality. Figure 3D) This graph provides an overview of the percentage of studies that used different imaging feature selection methods. Figure 3E) This graph provides an overview of the percentage of studies that investigated each type of trauma. Figure 3f) This graph is an overview of the number of literature with respective sample population; most studies include the sample size of 66 to 156. Figure 3G) This chart provides an overview of the types of tasks used by the task-based fMRI studies. The current review study found a total of 13 fMRI task-based studies; the figure demonstrates that the most used task is emotional picture task, followed by Go/NoGo task and Script-driven imagery task. The chart reveals that the tasks used are diverse, each task is used by 1 to 3 studies.

Figure 4: This figure illustrates the neural network alterations within the dissociative subtypes, specifically the difference in the directionality of connectivity within PTSD+DS vs PTSD-DS. Thick lines indicate two or more studies found consistent directionality of connectivity. Narrow line indicates the directionality of the connectivity is implicated by one study. Arrows illustrate the pattern of between-network connectivity; the square around the network names represent the pattern of within-network connectivity. Increased between-network connectivity is represented by red line and observed between the following networks: pDMN-brainstem, SN-pDMN, aDMN-Brainstem, ECN-BGN. Increased within-network connectivity observed in aDMN, ECN. Decreased network connectivity is represented by blue line, observed between the following networks: ECN-Brainstem. Inconsistent directionality of between-network. Decreased within-network connectivity was found in the following network: pDMN, SN, Cerebellar network, and Brainstem. Inconsistent network pattern, represented by grey dotted line, was found between the following networks: SN-Brainstem, Cerebellar-pDMN, ECN-aDMN, ECN-pDMN. Inconsistent within-network connectivity found in BGN. The network is defined as following brain hubs. pDMN: Posterior DMN, including PCC, precuneus, TPJ. aDMN: anterior DMN, including vmPFC. SN: salience network, including amygdala, insula. ECN: executive control network, including DLPFC, frontal pole, anterior cingulate cortex.
